# Microbiota modulation counteracts Alzheimer’s disease progression influencing neuronal proteolysis and gut hormones plasma levels

**DOI:** 10.1038/s41598-017-02587-2

**Published:** 2017-05-25

**Authors:** Laura Bonfili, Valentina Cecarini, Sara Berardi, Silvia Scarpona, Jan S. Suchodolski, Cinzia Nasuti, Dennis Fiorini, Maria Chiara Boarelli, Giacomo Rossi, Anna Maria Eleuteri

**Affiliations:** 1 School of Biosciences and Veterinary Medicine, University of Camerino, via Gentile III da Varano, 62032 Camerino, (MC) Italy; 2 Gastrointestinal Laboratory, Department of Small Animal Clinical Sciences, College of Veterinary Medicine and Biomedical Sciences, Texas A&M University, College Station, USA; 3 School of Pharmacy, Pharmacology Unit, University of Camerino, via Madonna delle Carceri, 62032 Camerino, (MC) Italy; 4 Division of Chemistry, School of Science and Technology, University of Camerino, I-62032 Camerino, MC Italy

## Abstract

Gut microbiota has a proven role in regulating multiple neuro-chemical pathways through the highly interconnected gut-brain axis. Oral bacteriotherapy thus has potential in the treatment of central nervous system-related pathologies, such as Alzheimer’s disease (AD). Current AD treatments aim to prevent onset, delay progression and ameliorate symptoms. In this work, 3xTg-AD mice in the early stage of AD were treated with SLAB51 probiotic formulation, thereby affecting the composition of gut microbiota and its metabolites. This influenced plasma concentration of inflammatory cytokines and key metabolic hormones considered therapeutic targets in neurodegeneration. Treated mice showed partial restoration of two impaired neuronal proteolytic pathways (the ubiquitin proteasome system and autophagy). Their cognitive decline was decreased compared with controls, due to a reduction in brain damage and reduced accumulation of amyloid beta aggregates. Collectively, our results clearly prove that modulation of the microbiota induces positive effects on neuronal pathways that are able to slow down the progression of Alzheimer’s disease.

## Introduction

Alzheimer’s disease (AD) is a common, progressive, and irreversible neurodegeneration with a gradual loss of memory, judgment, and ability to function. Treating and managing AD patients is a severe burden, and there is an urgent need to discover and validate new therapeutic agents. Most cases of early-onset AD derive from a combination of genetic mutations in genes encoding amyloid precursor protein (APP) and presenilins 1 and 2 (PSEN1 and PSEN2). APP cleavage by β and γ secretase complexes leads to the formation of amyloid-β (Aβ) peptides that can aggregate and form amyloid plaques, mainly composed of the 42 amino acid peptide (Aβ_1–42_), which is less abundant but more prone to aggregation than the Aβ_1–40_ peptide. Amyloid deposits and neurofibrillary tangles, comprising hyper phosphorylated tau protein, are the most important pathologic hallmarks of AD. Aβ deposition and clearance are finely regulated by the ubiquitin–proteasome system (UPS) and autophagy, which are tightly interrelated^1–4^. Impairment of proteolysis, which is characteristic of AD neurons, favors the accumulation of detrimental Aβ oligomeric structures that further contribute to proteasome and autophagy alterations.

Recently, several authors have described a role for gut peptide hormones in AD. These hormones are responsible for energy homeostasis and food intake regulation and show effects on the central nervous system (CNS), modulating nervous functions like learning and memory^5–7^. Ghrelin is involved in glucose and lipid metabolisms, but also in higher brain functions such as learning and memory; it influences mitochondrial respiration and exerts neuroprotective effects, takes part in the aetiopathogenesis of neurodegenerative disorders, representing a link between metabolism and neurodegeneration^8^. Ghrelin and leptin act as neurotrophic factors protecting cells against toxicity induced by Aβ oligomers^5, 9^. Plasma leptin concentration is negatively correlated to Aβ levels due to its direct regulatory effect on γ-secretase^10^. In addition, animal models of AD treated with leptin showed a reduction in Aβ and phosphorylated tau levels^11, 12^. The age-related decline in plasma ghrelin concentration and the impairment of the ghrelin signaling observed in AD patients is closely related to the compromised memory and learning processes^13^. The glucagon-like peptide 1 (GLP-1) protects cultured neurons form oxidative damage and formation of Aβ plaques, and controls synaptic plasticity in mice^14, 15^. The use of glucose-dependent insulinotropic polypeptide (GIP) analogs as neuroprotective agents is an emerging and promising strategy in the treatment of AD^16, 17^.

Currently, no definitive treatment exists for AD, and most approaches aim to preserve cognition and memory and to delay the loss of function. Recent studies have highlighted a role for the human microbiome in regulating multiple neuro-chemical pathways through the highly interconnected host-microbiome system, the so-called gut-brain axis^18–20^. Oral bacteriotherapy is becoming an accepted practice for the prevention and treatment of allergies^21^, gastrointestinal infections^22^, inflammatory conditions^23, 24^ and cancer^25^. Beneficial effects of lactic acid bacteria and bifidobacteria in CNS-related diseases such as multiple sclerosis, cognitive deficits, and stress-derived pathologies, have been recently documented^26–31^. Probiotic supplementation reverses cognitive impairment and ameliorates the spatial memory in diabetic rats^32^. It has recently been shown that the probiotic mixture VSL#3 modulates the expression of a number of genes in the brain cortex of aged rats, with positive consequences on inflammatory and neuronal processes^33^. Moreover, bacterial byproducts such as short chain fatty acids (SCFAs) exert a number of neuromodulator effects and directly act on gastrointestinal cells stimulating the synthesis of hormones such as leptin and GLP-1^34, 35^.

In the present study, a novel formulation of lactic acid bacteria and bifidobacteria (SLAB51) was administered to a triple-transgenic mouse model of Alzheimer’s disease, B6; 129-*Psen1tm1Mpm* Tg (APPSwe, tauP301L)1Lfa/J (named 3xTg-AD), in order to investigate the potential beneficial effects on memory deficits, amyloid plaque deposition, and neuronal proteolysis impairment. To gain insight into the effects of microbiota on AD progression the modulation of the gut-brain axis upon administration of the probiotic mix was also investigated.

## Methods

### Experimental design

8-week-old male 3xTg-ADmice (n = 64) were organized in two groups: a treated group (administered for 4 months with SLAB51 in water) and a control group (administered with water). Simultaneously, 64 coetaneous wild type (wt) mice were divided into wt control and wt treated groups. At 8, 12, 18 and 24 weeks of age, 15 animals per group were given the open field (OF) and novel object recognition (NOR) tests. Elevated plus maze and passive avoidance tasks were added for mice at 24 weeks of age. Mice were sacrificed for biochemical analyses at 12, 18 and 24 weeks of age. (Sample size for each group was 8). Eight additional AD mice and eight wt mice were sacrificed at 8-weeks of age representing time 0 for biochemical and immuno-histological analyses.

In a second experiment a new batch of both AD (n = 20) and wt (n = 20) animals has been treated with water (n = 10) or SLAB51 (n = 10) to perform the NOR test directly at 24 weeks of age. This second test was necessary to verify the hypothesis that 24-weeks-old mice could be saturated, and not motivated to explore any objects again, upon repeating the NOR for the fourth time.

### Availability of data and material

The data that support the findings of this study are available from University of Camerino but restrictions apply to the availability of these data, which were used under license for the current study, and so are not publicly available. Data are however available from the authors upon reasonable request and with permission of the University of Camerino.

## Experimental Procedures

### Reagents and chemicals

SLAB51 formulation was provided by Mendes Sa (Lugano, Switzerland). Substrates for assaying the chymotrypsin-like (ChT-L), trypsin-like (T-L), and peptidyl glutamyl-peptide hydrolyzing (PGPH) activities of the proteasomal complex were purchased from Sigma-Aldrich S.r.L. (Milano, Italy). The substrate Z-Gly-Pro-Ala-Leu-Ala-MCA to test the branched chain amino acids preferring (BrAAP) activity was obtained from Biomatik (Cambridge, Ontario). Aminopeptidase N (EC 3.4.11.2) for the coupled assay utilized to detect BrAAP activity^36^ was purified from pig kidney as reported elsewhere^37^. Cathepsin B and cathepsin L substrates (Z-Arg-Arg-AMC and Z-Phe-Arg-AFC.trifluoroacetate) were from Sigma-Aldrich S.r.L. (Milano, Italy). Membranes for western blotting analyses were purchased from Millipore (Milano, Italy). Proteins immobilized on films were detected with the enhanced chemiluminescence (ECL) system (Amersham Pharmacia Biotech, Milano, Italy). p27 antibody was purchased from Calbiochem (EDM Millipore, Billerica, MA). All the other antibodies were from Santa Cruz Biotechnology (Heidelberg, Germany). ELISA Kit for Aβ_1–40_ and Aβ_1–42_ peptide determination in brain homogenates were purchased from Invitrogen (Camarillo, CA). Proteases inhibitors tosyl phenylalanyl chloromethyl ketone (TPCK) and 4-(2-Aminoethyl) benzenesulfonyl fluoride hydrochloride (AEBSF or Pefabloc) were from Sigma-Aldrich S.r.L. (Milano, Italy).

### Animals

The triple-transgenic mouse model of AD, B6;129-*Psen1* ^*tm1Mpm*^ Tg (APPSwe,tauP301L)1Lfa/J (named 3xTg-AD) and their respective wild types mice were purchased from the Jackson Laboratory (Bar Harbor, Maine, USA). 3xTg-AD mice were previously characterized and represent a reliable model of human AD patients. In this model, Aβ intracellular immunoreactivity can be detected in some brain regions as early as three to four months of age^38^. Experiments were conducted using 8-week-old male mice (weight 15–25 g) in accordance with the guidelines laid down by the European Communities Council (86/609/ECC) for the care and use of laboratory animals. Mice were housed in plastic (Makrolon) cages (4 animals per cage) in a temperature controlled room (21 ± 5 °C) and 60% humidity on 12 h light/dark inverted cycle (light was switched on at 8:00 P.M.) and maintained on laboratory diet (Mucedola, Italy) with water ad libitum. All appropriate measures were taken to minimize pain and discomfort in experimental animals. Brains, livers, feces and urines were properly stored at −80 °C after sacrifice.

All procedures were in accordance with the guidelines laid down by the European Communities Council (86/609/ECC) for the care and use of laboratory animals under an approved protocol (EUFTP#261473) by Veterinary Health Dept. of the Italian Ministry of Health.

### SLAB51 administration

Two groups of mice (n = 32 animals for each group) were treated for four months as follows: a 3xTg-AD group orally treated with vehicle (water), a 3xTg-AD group orally treated with SLAB51, a formulation made of nine live bacterial strains (*Streptococcus thermophilus*, bifidobacteria (*B. longum, B. breve, B. infantis*), lactobacilli (*L. acidophilus, L plantarum, L. paracasei, L. delbrueckii subsp. bulgaricus, L. brevis*)). The dosage (200bn bacteria/Kg/day) was calculated by application of the body surface area principle^39^. Fresh drinking solution was changed every day. The body weight was measured every 2 weeks before starting the treatment and then once a month to ensure adequate intake of the experimental food.

### Behavioral assessments

All behavioural experiments were performed during the animal’s dark phase, with testing performed from 8:00 to 15:00. Animals were handled for 3 days before testing in order to accustom them to the experimenter. The investigators were blinded to the groups’ allocation during the tests.

The open field (OF) test was used to evaluate the locomotor activity of mice using automated locomotor activity boxes (Med Associates, VT 05478) as previously reported^40^. Locomotor activity was recorded for 5 mins, starting 1 min after placing the animal in the test cage. Each mouse was automatically recorded by interruptions of orthogonal light beams (3.5 cm above the activity box floor), which were connected to automatic software (Activity Monitor, Med Associates). The behavioral parameters observed were ambulatory (number of horizontal episodes) and stereotype counts (number of grooming movements).

The novel-object recognition (NOR) test is used to evaluate recognition memory and it is based on the spontaneous tendency of rodents to spend more time exploring a novel object than a familiar one. Mice were challenged in the open-field arena explored on the day before during OF. Following a training period, the animal was removed from the environment for a delay period of 3 h and then it was returned to the arena, where one of the two identical objects had been replaced by a new, dissimilar novel object (test phase). The amount of time the rodent spends exploring each object in 10 mins provides a powerful measurement of memory integrity and attention.

Results were expressed as discrimination score (seconds spent with novel object − seconds spent with familiar object)/(total time spent with both objects). Lower score indicates memory impairment in this task. Objects were different for shape, color and texture at each time point^41^ and maintained throughout the study to obtain reproducible data. Preliminary experiments were done to select novel and familiar object pairs on the basis that each object in the pairs elicited the same amount of spontaneous investigation.

The passive avoidance test is a fear-motivated test used to assess memory function based on the association formed between an aversive stimulus such as a mild foot shock and a specific environmental context. The amygdala plays a pivotal role in passive avoidance learning.

Apparatus and procedures were previously described^42^. Briefly, during the training test, each mouse received an electric shock when it entered the dark compartment. In the retention test, passive defensive reactions, assessed in terms of the latent period of transfer from the light to the dark compartment, were tested 24 h and 7 days after foot shock. Higher latency value translates to better retention of memory from the foot shock given during the learning phase^43^.

The elevated plus maze (EPM) is a test used to detect anxiety-related behavior in animals^44^. The apparatus, a cross-shaped wooden elevated maze, consisted of two opposite open arms 30 cm × 5 cm, and two opposite arms enclosed by 20-cm-high walls with two open arms and two closed arms. The maze was elevated 50 cm from the floor and lit by dim light. The procedure is described in Nasuti *et al*.^45^. Changes in the percentage of time spent and number of entries into the open arms indicate changes in anxiety-like behavior. A greater percentage of time spent and number of entries in open arms indicates less anxiety-like condition.

### Microbiota analysis

An aliquot of 100 mg (wet weight) of each fecal sample DNA was extracted with a DNA isolation kit (MoBio Power soil, MoBio Laboratoroies, USA) following the manufacturer’s instructions. The V4 region of the 16S rRNA gene was amplified with primers 515 F (5′-GTGCCAGCMGCCGCGGTAA-3′) and 806 R (5′-GGACTACVSGGGTATCTAAT-3′) at the MR DNA Laboratory (Shallowater, TX, USA) as previously described^46^. The Nextera® DNA sample Preparation kit including sequencing adapters and sample specific barcodes was used to prepare a DNA library and sequenced at MR DNA on an Illumina MiSeq instrument.

The raw sequences obtained were analyzed using the software QIIME v.1.8. A total of 5,343,083 were obtained. Sequences were demultiplexed, low quality reads were filtered using default parameters, chimeras removed and sequences were then clustered into operational taxonomic units (OTUs) using an open-reference OTU picking protocol at the 97% sequencing identity level against the Greengenes^47^ database. For further analysis, each was rarefied to an even sequencing depth of 24,800 sequences to adjust for uneven sequencing depth across all samples. Observed species richness, Chao 1, and Shannon indexes were determined using QIIME. The software PICRUSt (Phylogenetic Investigation of Communities by Reconstruction of Unobserved States) was used to make functional gene content predictions based on 16S rRNA gene data generated by all organisms found in the data and represented in the Greengenes phylogenetic tree of 16S rRNA gene sequences. Because most datasets did not meet the assumptions of normal distribution as assessed by the D’Agostino and Pearson normality test, non-parametric statistical tests were used. The Friedman test with Dunn’s post hoc test for repeated measures ANOVA was performed to evaluate changes among all timepoints. The resulting p-values were adjusted for multiple comparisons using the Benjamini & Hochberg’s False Discovery Rate (FDR), and an adjusted p < 0.05 was considered statistically significant. Data were analyzed using Prism software 5.0 (GraphPad Software, San Diego, CA) and JMP software (SAS Institute, Cary, NC, USA). Linear discriminant analysis effect size (LEfSe), freely available online in the Galaxy workflow framework, was used to elucidate taxa and genes associated with treatments at the various time-points. Analysis of beta-diversity was performed using unweighted Unifrac distance metrics. Statistical significance of the resulting distance metric was tested by analysis of similarities (ANOSIM) using the QIIME software. Sequences were deposited in the SRA archive under the accession number: SRP064106. The software PICRUSt (Phylogenetic Investigation of Communities by Reconstruction of Unobserved States) was used to predict the functional gene content in the fecal microbiome based on the 16S rRNA genes found in the data and represented in the Greengenes phylogenetic tree of 16S rRNA gene sequences. PICRUSt was used online in the Galaxy workflow framework. Linear discriminant analysis effect size (LEfSe) was used to elucidate bacterial taxa (16S rRNA genes) and functional genes (PICRUSt) associated with healthy or diseased cats.

### Short chain fatty acids (SCFAs) determination

Fecal content of acetic, propionic and butyric acid has been quantified by means of headspace solid-phase microextraction coupled to gas chromatography with flame ionization detection by using a polydimethylsiloxane/carboxen/divinyl benzene coated fiber, following the procedure by Fiorini *et al*.^48^. Data are expressed as mean content (mmol/Kg) ± SD and were statistically analyzed using one-way analysis of variance, followed by the Tukey-Kramer method for post-hoc analysis. Different superscript letters (a, b) indicate significant variations at P < 0.05 in the table.

### ELISA assay for ghrelin, leptin and GIP, GLP-1

Plasma hormone concentrations were measured through ELISA using plasma treated with protease inhibitors (Pefabloc and TPCK).

Briefly, the Rat/mouse Ghrelin Active ELISA kit is a sandwich ELISA based on the capture of ghrelin molecules (active form) in the plasma by anti-ghrelin IgG and the immobilization of the resulting complex to the wells of a microtiter plate coated by a pre-titered amount of anchor antibodies. After the binding of a second biotinylated antibody to ghrelin and the wash away of unbound materials, followed by conjugation of horseradish peroxidase to the immobilized biotinylated antibodies, the quantification of immobilized antibody-enzyme conjugates is performed by monitoring horseradish peroxidase activities in the presence of the substrate 3,3′,5,5′-tetra-methylbenzidine. The enzyme activity is measured spectrophotometrically by the increased absorbency at 450 nm, corrected from the absorbency at 590 nm, after acidification of formed products. Since the increase in absorbency is directly proportional to the amount of captured rat/mouse ghrelin (active form) in the unknown sample, the concentration of active ghrelin can be derived by interpolation from a reference curve generated in the same assay with reference standards of known concentrations of rat/mouse ghrelin.

Leptin and GIP were determined using sandwich ELISA kit based on anti-leptin and anti-GIP monoclonal antibodies respectively.

Similarly, the quantitative determination of mouse glucagon like peptide-1 was performed using a sandwich ELISA kit (CUSABIO Cat #CSB-E08118m). Antibody specific for GLP-1 was pre-coated onto a microplate. Standards and samples are pipetted into the wells and any GLP-1 present is bound by the immobilized antibody. After removing any unbound substances, a biotin-conjugated antibody specific for GLP-1 is added to the wells. After washing, avidin-conjugated horseradish peroxidase is added to the wells. Following a wash to remove any unbound avidin-enzyme reagent, a substrate solution is added to the wells and color develops in proportion to the amount of GLP-1 bound in the initial step. The color development is stopped and the intensity of the color is measured.

### Cytokine analyses

The plasma levels of pro- and anti- inflammatory cytokines were measured through ELISA using the Mouse Inflammatory Cytokines & Chemokines Multi-Analyte ELISArray Kit (QIAGEN, Italy). Samples and standards were prepared following the manufacturer’s protocols. Each cytokine level was calculated based on its own standard curve and expressed as mean concentration (pg/ml) ± SE.

### Congo red staining and immunohistochemistry analysis

Three 3 μm-thick parasagittal sections from each animal (n = 8 per sub-group), at ~0.84, 1.20, and 1.56 mm lateral from the midline^49^, were prepared. Selected sections were deparaffinized and rehydrated according to standard protocols, and they were used for Congo red staining and for Aβ and FGF9 immunohistochemical detection. Hematoxylin and eosin counterstaining was used to provide morphological details.

In detail, Aβ peptides were immunodetected using a polyclonal antibody with specificity for the Aβ_1–42_ C-terminus (Millipore, CA). Briefly, for each time point (8, 12, 18, 24 weeks of age), brain slides from treated and untreated wt and AD mice (n = 8 per subgroup) were fixed in a 50:50 mixture of methanol and acetone for 5 min and incubated with the anti-Aβ_1–42_ antibody (1:50). The binding of the antibody was detected with the Elite kit (Vector Laboratories), and the immunoreaction was developed using diaminobenzidine chromogen (DAB, Vector).

For FGF9 detection, brain sections were incubated overnight with anti-FGF9 rabbit polyclonal antibody (aa50–99) IHC-plus™ (LSBio Catalog No. LS-B11953), diluted 1:50, cross reacting with mouse, human, bovine and other animal species. Non-specific binding was blocked by incubation of slides for 10 minutes with a protein-blocking agent (Protein-blocking agent, Dako, Carpinteria, CA, USA) before application of the primary antibody. Slides were incubated overnight in a moist chamber. The immunoreaction with streptavidin–immunoperoxidase (Streptavidin–immunoperoxidase, Black & Decker, Towson, MD, USA) was visualized with 3,3′-diaminobenzidine substrate (3,3′-diaminobenzidine substrate, Vector, Burlingame, UK). Tissues were counterstained with Mayer’s hematoxylin. For negative immunohistochemical controls the primary antibodies were omitted. Sections of human and bovine brain, AD and BSE respectively affected, served as positive control tissues for Aβ_1–42_ and FGF9 cell staining. For scoring of Congo red, Aβ_1–42_, and FGF9 positive cells, these cells were quantified in different area of the mouse brain, particularly select compartments of the CNS as hippocampal area. All cellular types were evaluated using a light microscope (Carl Zeiss, Jena, Germany), a 40x objective, a 10x eyepiece, and a square eyepiece graticule (10 × 10 squares, having a total area of 62,500 μm^2^). Ten appropriate fields were chosen for each compartment and arithmetic means were calculated for each brain region. Results were expressed as IHC positive cells per 62,500 μm^2^. For all parameters, cells on the margins of the tissue sections were not considered for evaluation to avoid inflation of positive cell numbers. Positive Congo red/Aβ_1–42_/FGF9 cells, amyloid interstitial plaques, and other CNS areas, were quantified by using an image-analysis system consisting of a light microscope (Carl Zeiss, Jena, Germany) attached to a Javelin JE3462 high-resolution camera and a personal computer equipped with a Coreco-Oculus OC-TCX frame grabber and high-resolution monitor. Computerized color-image analysis was performed by using Image-Pro Plus software (Media Cybernetics). The entire cerebral cortex and hippocampus were separately sampled with the counting frame size 250 μm × 250 μm for cortex and 100 μm × 100 μm for hippocampus. The area of each section in all cross brain sections in every mouse was recorded, as was the total number of neurons determined by immunostaining as previously described. For each mouse, the total brain area was calculated as the sum of the areas of all fields in all brain cross sections on one slide. Congo red, Aβ_1–42_, and FGF9 positive cells were counted per section, and stained cell densities were expressed as the number of cells per square millimeter of analyzed section area^24^. The sum of the area of all amyloid plaques was divided by the total area of cerebral cortex or hippocampus to obtain the amyloid burden. The pathologist performing quantification of amyloid burden was blind to age, treatment type, and genotype of mice. The unbiased stereological based quantification of amyloid burden was performed on the basis of methodology suggested by Liu *et al*.^50^.

### Measurement of the cortex

For the thickness measurement of the cortex wall, serial sections, in the coronal plane, from rostral to caudal of each mouse cerebrum were made. Briefly, the brains were removed, placed in 10% buffered formalin, covered with aluminum foil, and refrigerated. Two-mm-thick sections were cut 4, 6, and 8 mm from the frontal pole, and sections were photographed within 24 h. To prevent distortion, brain slices were kept flat in the Petri dish overnight. After the sections had been photographed, they were routinely processed and paraffin embedded, then newly 3 µm sectioned and stained with hematoxylin and eosin (H&E) and newly photographed. The serial sections were sub-divided into frontal, parietal, temporal, and occipital regions of the brain in rostral to caudal direction. Qualitative and quantitative analyses of the wall of the cerebral hemisphere were carried out on every 20th section. For each sub-group, 5 sections were analyzed. In each of these coronal sections, brains were stained with H&E and the cortex thickness was measured. For the assessment of regional cortical atrophy the thicknesses of the wall and laminae was determined by thickness measurements on H&E-stained sections. The different zones were measured using a calibrated integrating graticule (0.01mm) in a single eyepiece. The method of point counting was used to determine the relative volume proportion of the cerebral wall, meninges and ventricles. The same stratified selected sections were projected onto a screen using a Leitz demonstration microscope (x2.5). A grid with 300 points was superimposed on the image. The points falling on the cerebral wall, meninges, and ventricle were counted field-by-field to cover the entire left and right cerebral hemispheres.

### Ventricular sizes evaluation in the brain sections

Equivalent sections of 3xTg-AD, and wild-type mice brains were chosen on the basis of common morphological landmarks^49^. Images of stained tissues were converted into TIFF format using Adobe Photoshop Elements 2.0. The areas of the brain substance and ventricles were measured using NIH ImageJ 1.39 u. Two images from each individual mouse were considered.

### TUNEL analysis

In brain sections, apoptotic index was highlighted through a TUNEL colorimetric staining (DeadEnd, Promega®) according to the manufacturer’s instructions. For evaluation of the apoptotic rate, ten random fields of any chamber were examined under a dry- 40x objective. TUNEL-positive cells are characterized by a brownish-black nuclear stain. Lower-power digitized images were acquired with a light microscope (Carl Zeiss, Jena, Germany) attached to a Javelin JE3462 high-resolution camera and a personal computer equipped with a Coreco-Oculus OC-TCX frame grabber and high-resolution monitor, and cells count and quantification were performed as previously reported.

### Preparation of brain extracts

Brain extracts were homogenized (1:5 weight/volume of buffer) in 50 mM Tris buffer, 150 mM KCl, 2 mM EDTA, pH 7.5. Homogenates were immediately centrifuged at 13.000 × *g* for 20 min at 4 °C and the supernatant was collected for enzymes activity assays and western blotting. A small part of this supernatant fraction was immediately supplemented with protease inhibitors for hormones determination by ELISA as described below. Protein content was determined by the Bradford method^51^ using bovine serum albumin (BSA) as standard.

### Proteasome activity assays

Proteasome peptidase activities in brain homogenates (supernatant fraction) were determined using synthetic fluorogenic peptides: Suc-Leu-Leu-Val-Tyr-AMC was used for ChT-L activity, Z-Leu-Ser-Thr-Arg-AMC for T-L activity, Z-Leu-Leu-Glu-AMC for PGPH activity, and Z-Gly-Pro-Ala-Phe-Gly-pAB for BrAAP activity^52^. The incubation mixture contained brain homogenates (15 μg total proteins), the proper substrate (5 μM final concentration) and 50 mM Tris–HCl pH 8.0, up to a final volume of 100 μL. Incubation was performed at 37 °C for 60 min and the fluorescence of the hydrolyzed 7-amino-4-methyl-coumarin (AMC) and 4-aminobenzoic acid (pAB) was detected (AMC, λ_exc_ = 365 nm, λ_em_ = 449 nm; pAB, λ_exc_ = 304 nm, λ_em_ = 664 nm) on a SpectraMax Gemini XPS microplate reader. The 26S proteasome ChT-L activity was tested including in the reaction mix 10 mM MgCl_2_, 1 mM dithiothreitol, and 2 mM ATP.

### Cathepsin B and L

Cathepsin B and L proteolytic activities were measured following the protocol described by Tchoupè *et al*.^53^ using the fluorogenic peptides Z-Arg-Arg-AMC and Z-Phe-Arg-AFC, respectively, at a final concentration of 5 μM. The mixture for cathepsin B, containing 7 μg of protein lysate, was pre-incubated in 100 mM phosphate buffer pH 6.0, 1 mM EDTA and 2 mM dithiothreitol for 5 min at 30 °C. Upon the addition of the substrate, the mixture was incubated for 15 min at 30 °C. The mixture for cathepsin L, containing 7 μg of protein lysate, was incubated in 100 mM sodium acetate buffer pH 5.5, 1 mM EDTA and 2 mM dithiothreitol for 5 min at 30 °C and, upon the addition of the substrate, the mixture was incubated for 15 min at 30 °C. The fluorescence of the hydrolyzed 7-amino-4-methyl-coumarin (AMC, λexc = 365 nm, λem = 449 nm) and 7-amino-4-trifluoromethylcoumarin (AFC, λexc = 397 nm, λem = 500 nm) was detected on a SpectraMax Gemini X PS microplate reader.

### Western blotting analyses

Brain homogenates (supernatant fraction) were analyzed through western blotting assays with the aim to measure the following intracellular protein levels: amyloid oligomers, ubiquitinated proteins, p53, p27 and the autophagy related proteins Beclin-1, p62 and LC3-II. In detail, for each time point brain homogenates (20 μg total protein) were loaded on 12% SDS-PAGE (15% for LC3; 10% for ubiquitinated proteins and amyloid oligomers) and electroblotted onto PVDF membranes. Successively, upon incubation with specific antibodies, the immunoblot detections were carried out with Enhanced ChemiLuminescence western blotting analysis system (Amersham Pharmacia-Biotech). Molecular weight markers (6.5 to 205 kDa) were included in each gel. Glyceraldehyde-3-phosphate dehydrogenase (GAPDH) was used to check equal protein loading. The bands were quantified by using a densitometric algorithm. Each Western Blot was scanned (16 bits greyscale) and the obtained digital data were processed through Image J (NIH)^54^ to calculate the background mean value and its standard deviation. The background‐free image was then obtained subtracting the background intensity mean value from the original digital data. The integrated densitometric value associated to each band was then calculated as the sum of the density values over all the pixels belonging to the considered band having a density value higher than the background standard deviation. The band densitometric value was then normalized to the relative GAPDH signal intensity. The ratios of band intensities were calculated within the same Western Blot. All the calculations were carried out using the Matlab environment (The MathWorks Inc., MA, USA)^55^.

### ELISA assay for Aβ levels determination

Brain homogenates (supernatant fraction) promptly supplemented with protease inhibitors (Pefabloc and TPCK) were used to measure Aβ_1–40_ and Aβ_1–42_ levels using enzyme-linked immunosorbent assay NOVEX^®^ ELISA kits (Invitrogen,). Based on preliminary tests, samples were diluted at 1:5 with diluent buffer provided by the kit. Assays were performed according to the manufacturer’s directions.

### Statistical analysis

Results of behavioral tests were expressed as mean ± S.E. In particular, the EPM was analyzed by mean of Student’s t test. For the OP, NOR and passive avoidance, a two-way ANOVA with one factor within (time) and one factor between (treatment) was employed and appropriate post-hoc analysis was carried out using the Newman-Keuls test. Biochemical and IHC data are expressed as mean values ± S.E. Statistical analysis was performed with one way ANOVA, followed by the Bonferroni test using Sigma-stat 3.1 software (SPSS, Chicago, IL, USA). P-Values p < 0.05 were considered to be significant.

## Results

### Administration of SLAB51 counteracts cognitive decline and brain damage in AD mice

The effect of the probiotic on the consolidation process of memories in AD mice was assessed through the novel object recognition (NOR) and the passive avoidance tests, which have been used as cognitive probes for detecting hippocampus and amygdala functions, respectively.

**Figure 1:**
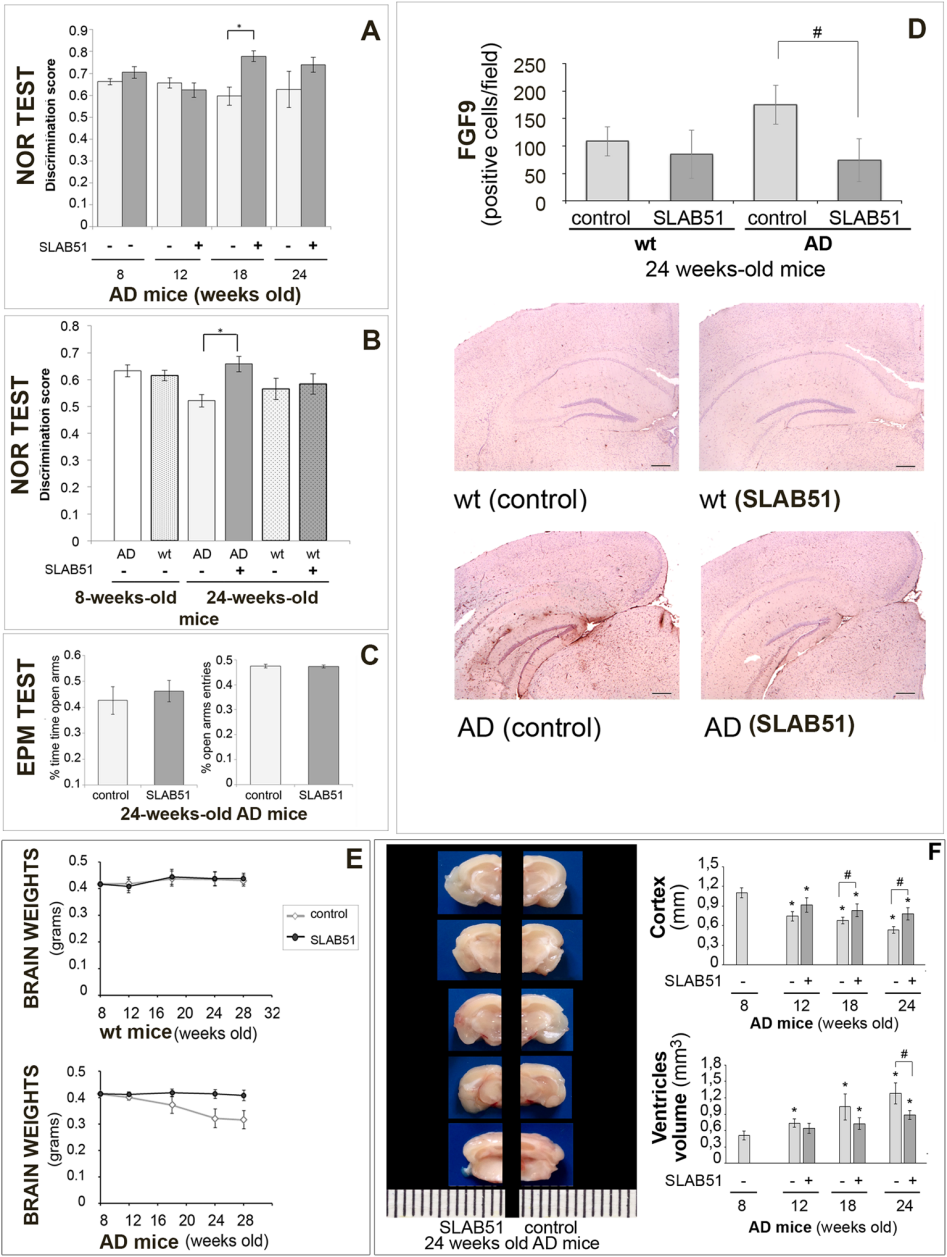
SLAB51 ameliorates behavioral performance and reduces brain damage in AD mice. *Novel Object Recognition (NOR) test* (first experiment): 15 mice/sub-group were allowed to explore an identical pair of objects, and after 3 hours, they are presented with the familiar object and a novel object. The discrimination scores for 8, 12, 18, and 24-week-old AD mice are reported in panel A. *NOR test* (second experiment) performed for the first time on treated and untreated 24-week-old mice (groups’ size = 10), panel B. Panel C: *Elevated plus maze test*. % open arm entries and % time spent in the open arms by untreated and treated AD mice at 24 weeks of age (first experiment, groups’ size = 15). Data points marked with an asterisk are statistically significant compared to their respective non-treated control mice (*p < 0.05). Panel D Immunodetection of FGF9 protein in brain slides of 24-weeks old untreated and treated wt and AD mice (8 animals per group) from the first experiment. Results are reported as number of cells immunoistochemically positive for FGF9 per field ± ES (^#^statistically significant with respect to the corresponding untreated mice p < 0.05). For each histological section 5 randomly selected field were analyzed at 40xHPFs. Representative images of immunohistochemical staining are reported. Panel E: Brain weights expressed in grams ± ES of both treated and untreated wt and AD mice over time (groups’ size is 8). Panel F: Measurement of the cortex thickness (mm) and ventricular sizes evaluation (mm^3^) in the brain sections of control and SLAB51 treated AD mice at 8, 12, 18 and 24 weeks of age. Data are reported as mean values ± ES (*statistically significant with respect to 8 weeks-old untreated mice p < 0.05; ^#^statistically significant with respect to the corresponding untreated mice p < 0.05). Consecutive brain slides of treated and untreated 24 weeks-old AD mice are shown.

In a first experiment, the AD mice were submitted to the NOR test at all time points (weeks 8, 12, 18 and 24) and differences between treated and control mice were observed (F[1,28] = 6.8, p < 0.05) as shown in Fig. 1, panel A. In particular, AD mice treated with the probiotic showed better “discrimination index” than untreated animals at 18 weeks of age, suggesting the beneficial effect of SLAB51 after 10 weeks of treatment. However, we could not see differences between 24-week-old treated and untreated mice. It is likely that mice may be not motivated to explore any objects again, regardless of whether they are familiar or novel. Indeed, compared to 18-week-old mice (25 s and 19 s for treated and untreated mice, respectively), the 24-week-old ones spent significantly less total time exploring both objects during the test phase (16.8 s and 9.6 s for treated and treated mice, respectively). To verify this hypothesis, a second group of AD and wt mice were submitted to the NOR test at only two time points (weeks 8 and 24). Here, we could appreciate differences between treated and untreated AD mice when they were 24 weeks old (F[3,40] = 2.95, p > 0.05) as shown in Fig. 1, panel B. In particular, treated AD mice showed an improvement in cognitive performance compared to age-matched untreated AD mice, demonstrating a sustained beneficial effect of SLAB51 until 24 weeks of age. No significant differences were observed between treated wt mice and age-matched untreated wt mice.

In the first experiment, 24-week-old treated and untreated AD mice underwent the passive avoidance test. Statistical analysis computed on passive avoidance behavior of AD mice showed no significant differences in the test performed at 24 h and 7 days (F[1,27] = 0.55, p > 0.05) after the training test, whereas a significant effect of time (F[2,54] = 117.84, p < 0.05) was observed (data not shown). However, higher entry latencies near the cut-off value (300 s), measured in the retention test, demonstrated that AD mice were able to memorize the punishment and to perform the inhibitory avoidance, indicating that triple mutation did not impair amygdala function.

24-week-old treated and untreated AD mice, from the first experiment, were evaluated in the elevated plus maze (EPM) to analyze their anxiety-like behavior. Statistical tests computed on the % open arm entries (df = 28, t = 0.30, p > 0.05) and on the % time spent in the open arms (df = 28, t = −1.08, p > 0.05) revealed no significant between-group differences, reflecting the same level of anxiety-like responses in treated and untreated age-matched AD mice (Fig. 1, panel C).

To verify that the beneficial effects of SLAB51 on attenuating cognitive impairment were not linked to an increased locomotor activity, AD mice (from the first experiment) were evaluated in an open field (OF) test at each time point (weeks 8, 12, 18 and 24). Two-way ANOVA revealed no differences in locomotor activity between treated and control AD groups (ambulatory counts: F[1,28] = 0.116, p > 0.05; stereotypic counts: F[1,28] = 1.95, p > 0.05) as shown in Table 1. These data suggested that SLAB51 treatment did not influence the locomotor activity of AD mice. Together, these findings rule out the possibility that, in the NOR, the different response between treated and control AD mice could depend upon differences in locomotor activity.

**Table 1:** SLAB51 effect on locomotor activities.

Weeks	8	12	18	24
**Ambulatory counts**				
AD control	571 ± 52.82	340 ± 55.16	384 ± 10.48	297 ± 8.53
AD + SLAB51	556 ± 80.92	367 ± 49.19	351 ± 14.55	298 ± 15.31
**Stereotypic counts**				
AD control	416 ± 12.60	378 ± 18.69	350 ± 13.60	313 ± 20.58
AD + SLAB51	410 ± 13.93	343 ± 21.83	325 ± 20.67	302 ± 19.47

*Open Field test*. Locomotor activities were registered after 5 mins. Ambulatory and stereotypic counts are expressed as means ± SEM. Group sizes: AD control (n = 15), AD + SLAB51 (n = 15). (p > 0.05).

These results are consistent with the expression levels of FGF9 in the hippocampal areas of 24 week-old mice (4 months of treatment), as reported in Fig. 1, panel D. Based on published data indicating that specific growth factor transcripts are altered in depressed, stressed, and AD-affected brains^56–58^, we selectively examined FGF9 differential expression in AD and wt mice. Results indicate a significant difference in FGF9 expression between treated and untreated AD mice, and a similar level and pattern of expression between treated AD and control wt mice. In fact, hippocampal FGF9 expression was consistently increased in untreated AD mice compared to treated AD and control wt mice. Thus, these analyses confirmed the alteration of FGF9 in depressive disorders, and show also its up-regulation in the altered hippocampus of AD-affected mice. Few studies have previously demonstrated the increased FGF9 expression in the frontal cortices^56^ and locus coeruleus^57^ in patients with major depression. Moreover, so far no data are available on the role of FGF9 in the hippocampus during AD or its role in the regulation of emotionality. Thus, our results could be helpful in elucidating the potential role of the hippocampal FGF9 in conditioning emotions and behavioral performances in AD.

The body weight of mice was controlled during the entire period of treatment and no differences were obtained between control and AD animals, indicating that the mixture was well tolerated. Interestingly, the brain weight of SLAB51-treated mice showed no changes, whereas in control animals a significant decrease over time was observed (Fig. 1, panel E).

Regarding brains morphology, it was immediately apparent that lateral ventricles were enlarged in 3 × Tg-AD untreated mice compared with SLAB51 treated animals (Fig. 1, panel F). In some areas of the brain, notable differences in the cortex thickness were observed between treated and untreated AD mice. Differences in thickness of the cerebral cortex were also minimal at t0 (8 weeks-old) and t1 (12 weeks-old), though the ventricular zone appeared slightly enlarged in untreated AD mice. Starting from t2 (18 weeks-od), the difference in cortical thickness became more pronounced between the two groups (Fig. 1, panel F) in particular in the hippocampal area (bregma −2.18 mm), at level of interventricular foramem (bregma −0,22 mm), and lateral ventricle at the point of alignment with the anterior arm of corpus callosum (bregma 0.62 mm). These data suggest the beneficial effect of SLAB51 in counteracting the decline of cortical thickness and the ventricular dilatation that are typical damages in AD brains.

### SLAB51 modifies intestinal microbiota

**Figure 2:**
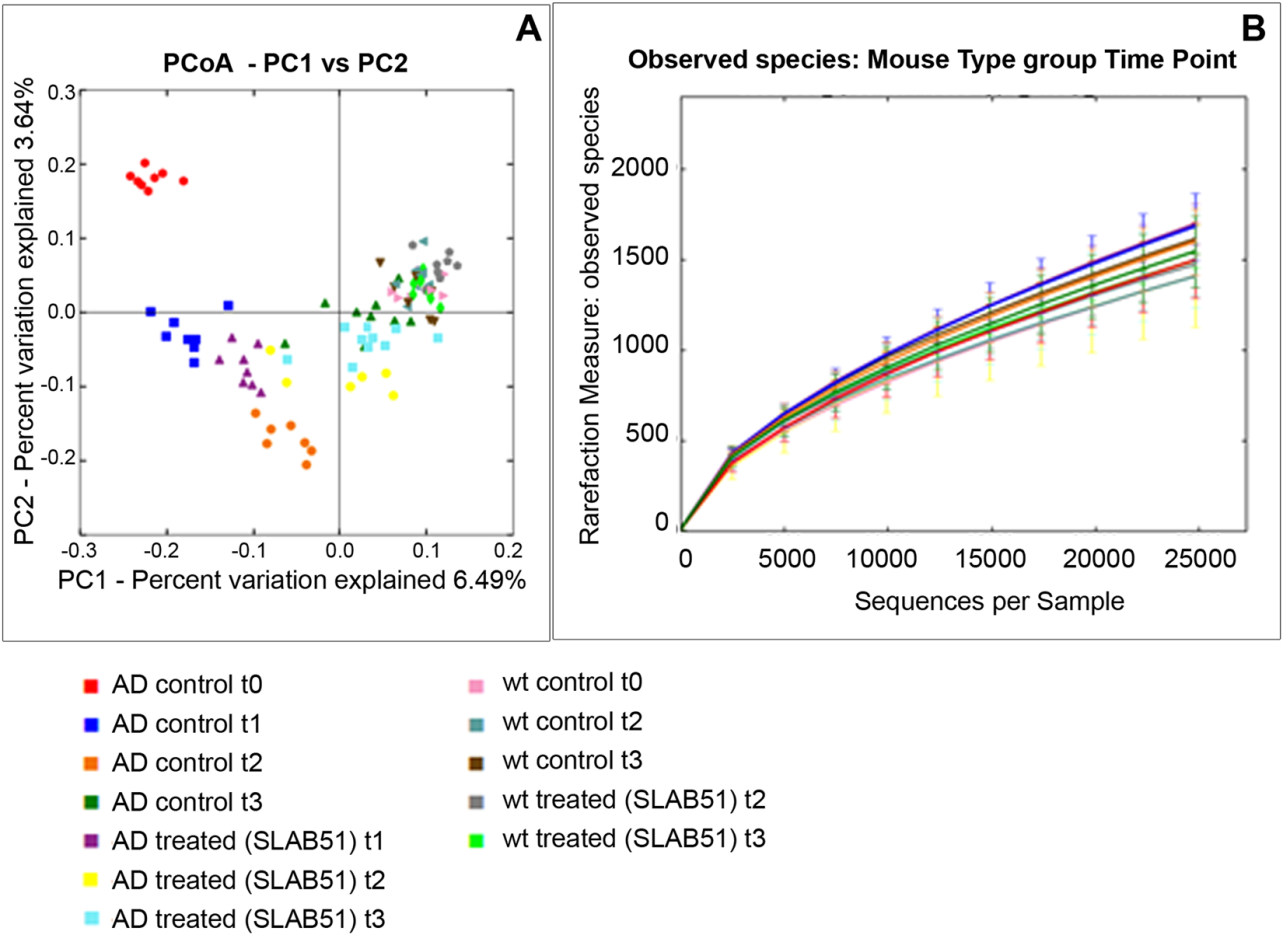
Microbiota analysis. PCoA plots based on unweighted Unifrac distances and rarefaction curves are presented. The microbiota was analyzed using 16S rRNA gene sequencing. PCoA plots based on unweighted Unifrac distances reveal separation between the microbiota of wt and AD mice at all time points, indicating that the affected mice have different microbiota structure compared to wt mice. No significant differences were found in alpha diversity indices, revealing no difference in species richness. The progression over time from week 8 to week 24 was more pronounced in the AD mice compared to the wt mice. Also, treatment with SLAB51 induced larger changes in AD mice compared to wt mice, but no differences in species richness (t0 = 8 weeks; t1 = 12 weeks; t2 = 18 weeks; t3 = 24 weeks).

PCoA plots of unweighted Unifrac distances revealed significant differences in microbiota structure between the wt and 3xTg-AD mice at all time points, but no significant differences were observed in species richness between wt and AD mice (Fig. 2). Considering untreated mice, lower concentrations of *Tenericutes*, *Cyanobacteria*, *Anaeroplasmatales, and Anaerostipes* were present in AD mice with respect to wt mice, with *Anaerostipes* playing an important role in gut health for the ability to produce butyric acid^59^ (Supplemental Table S1). Furthermore, the microbiota structure underwent more changes over the various time points in the 3xTg-AD, as the distances in the PCoA plots were larger, while the distances between the weeks clustered closer together in the wt mice. Moreover, treatment with SLAB51 induced larger changes in AD mice compared to wt mice. However, after adjustment for multiple comparisons, only a few bacterial taxa were found to be significantly different when either group of mice were or were not treated with SLAB51 (Supplemental Tables S2 and S3). Overall, similar changes in specific taxa were observed between the animal groups, and most notably, an increase in *Bifidobacterium spp*. and a reduction in *Campylobacterales* (i.e., *Helicobacteriaceae*; p = 0.04) was observed in both mouse groups when treated with SLAB51. The predicted functional bacterial metagenome content using PICRUSt revealed different effects of SLAB51 between wt and 3xTg-AD mice. While 14 pathways were increased (LDA score >4.0) due to SLAB51 in the 3xTg-AD mice, only one pathway was increased in wt mice (Table 2). SLAB51 induced several metabolic pathways in the 3xTg-AD mice, including DNA repair, pyrimidine metabolism, transcription machinery, energy metabolism, and glycolysis-gluconeogenesis.

**Table 2:** Effects of SLAB51 on KEGG orthologs in 24 weeks old wt and 3xTg-AD mice.

KEGG orthologs	LDA score	Group
Two-component system	4.20	3xTg-AD not treated
Bacterial motility proteins	4.13	3xTg-AD not treated
Secretion system	4.10	3xTg-AD not treated
General function prediction only	4.55	3xTg-AD treated SLAB51
DNA repair and recombination proteins	4.47	3xTg-AD treated SLAB51
Pyrimidine metabolism	4.31	3xTg-AD treated SLAB51
Peptidases	4.30	3xTg-AD treated SLAB51
Chromosome	4.21	3xTg-AD treated SLAB51
Amino sugar and nucleotide sugar metabolism	4.17	3xTg-AD treated SLAB51
Ribosome biogenesis	4.15	3xTg-AD treated SLAB51
Methane metabolism	4.14	3xTg-AD treated SLAB51
DNA replication proteins	4.13	3xTg-AD treated SLAB51
Alanine aspartate and glutamate metabolism	4.06	3xTg-AD treated SLAB51
Energy metabolism	4.04	3xTg-AD treated SLAB51
Glycolysis-gluconeogenesis	4.02	3xTg-AD treated SLAB51
Transcription machinery	4.01	3xTg-AD treated SLAB51
Homologous recombination	4.01	3xTg-AD treated SLAB51
Function unknown	4.13	wt not treated
Other ion-coupled transporters	4.07	wt treated SLAB51

**Figure 3:**
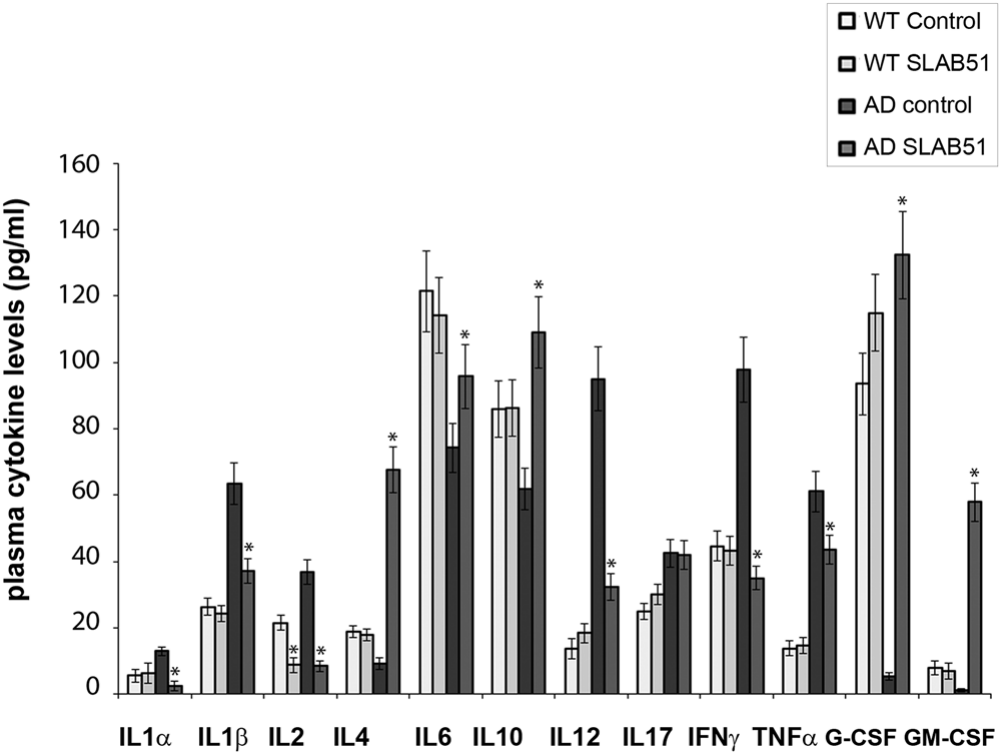
Inflammatory cytokines. ELISA of inflammatory cytokines measured in the plasma of 24 week-old wt and AD mice untreated or treated with SLAB51. Analytes concentrations are expressed as mean ± SE. Data points marked with an asterisk are statistically significant compared to their respective untreated mice (*p < 0.05).

Additionally, the fecal content of SCFAs was evaluated considering that these bacterial byproducts are known to act on the brain and that may mediate the effect on AD pathology. Interestingly, Table 3 shows that acetic, propionic and butyric acids significantly increased in AD mice upon SLAB51 treatment. Considering that the composition of gut microbiota and its metabolites can influence the inflammatory signaling, a panel of pro- and anti- inflammatory cytokines has been evaluated in the plasma of both wt and AD mice administered with water or SLAB51.Upon SLAB51 administration reduced plasma concentrations of pro-inflammatory cytokines have been observed in AD mice (Fig. 3), confirming that the modified microbiota produced anti-inflammatory effects. In detail, higher plasma concentrations of pro-inflammatory cytokines such as IL1α, IL1β, IL2, IL12, IL17, IFNɣ, and TNFα were observed in untreated AD mice with respect to wt counterpart. Interestingly, upon SLAB51 treatment a significant decrease of IL1α, IL1β, IL2, IL12, IFNɣ, and TNFα occurred. Moreover IL4, IL6, G-CSF, and GM-CSF, are less concentrated in the plasma of untreated AD mice with respect to wt mice. The probiotic treatment resulted in an increase of these cytokines that can down-regulate inflammatory response^60^. Collectively, these data are in agreement with the enriched gut content of the recognized anti-inflammatory SCFAs in AD mice upon probiotics supplementation.

**Table 3:** Short chain fatty acids (SCFA) fecal content (mmol/Kg) ± standard deviation in 24 weeks old 3xTgAD mice.

	Acetic acid	Propionic acid	Butyric acid	Total SCFA
	(mmol/Kg)			
AD control	26.64^a^ ± 5.66	8.53^a^ ± 2.91	4.15^a^ ± 4.15	39.31^a^ ± 11.61
AD + SLAB51	42.62^b^ ± 2.23	13.65^b^ ± 3.30	15.52^b^ ± 7.81	71.79^b^ ± 12.03

Different letters within the same column indicate significant differences (P < 0.05, one-way analysis of variance and Tukey’s test for pairwise comparison).

### Increased gut hormone concentration with SLAB51 treatment

**Figure 4:**
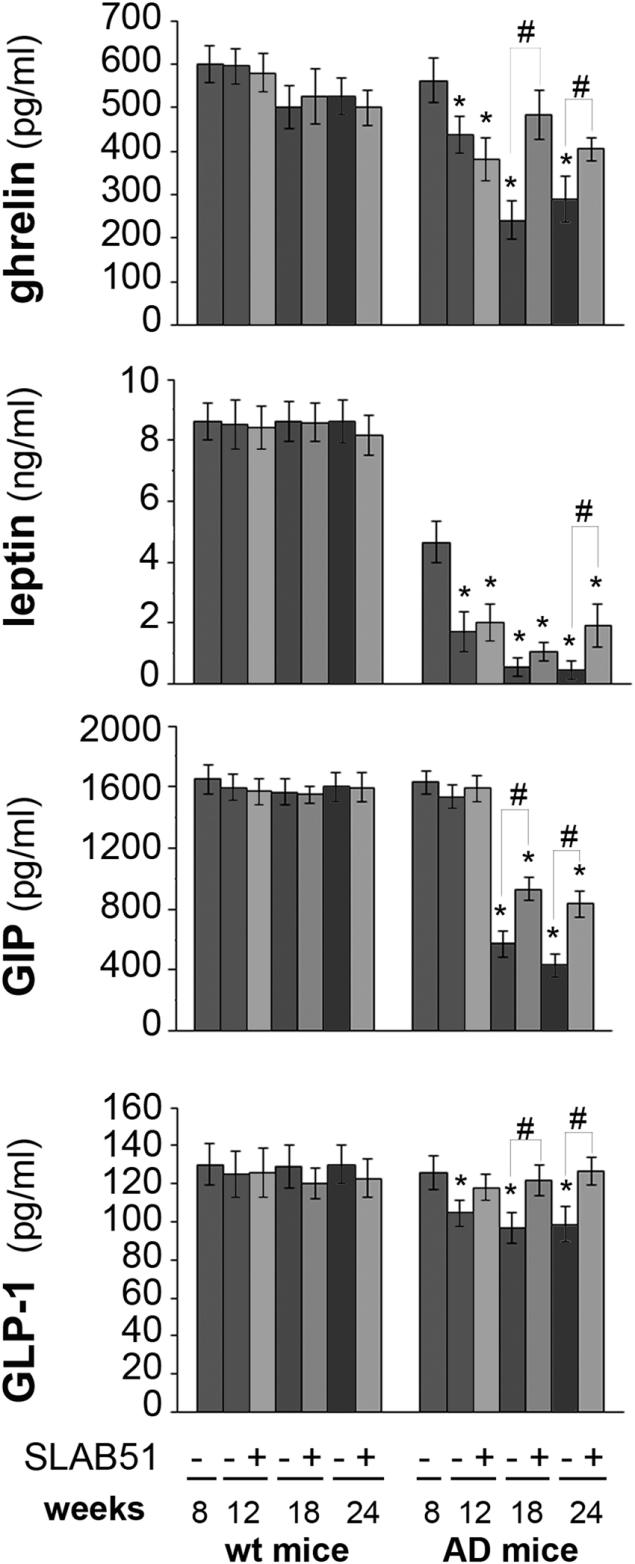
Plasma concentrations of gut hormones. Hormones were determined in the plasma of treated and control wt and AD mice. Results are expressed as percentage with respect to 8 week-old untreated mice. Data points marked with an asterisk are statistically significant compared to 8 week-old untreated control mice (*p < 0.05). Data points marked with hash are statistically significant compared to their respective control mice in the same time point (^#^p < 0.05).

We measured the plasma concentration of the gut peptide hormones ghrelin, leptin, GLP-1 and GIP because of their neuroprotective effects and potential as therapeutic targets. No changes in hormone plasma levels were observed in treated wt mice compared to the respective controls. An age-dependent decrease of the levels of all the tested hormones was observed in untreated AD mice. Interestingly, treatment with the probiotic formulation specifically increased the plasma concentration of these hormones. Results indicate significantly increased levels of ghrelin and GIP in 18- and 24-week-old treated AD mice compared to their respective controls. The level of leptin upon SLAB51 administration increased in treated 24-week-old AD mice compared to untreated animals. Treatment with SLAB51 induced an increase in GLP-1 plasma concentration by 12 weeks of age (Fig. 4).

### SLAB51 decreased amyloid load in AD mice brain

**Figure 5:**
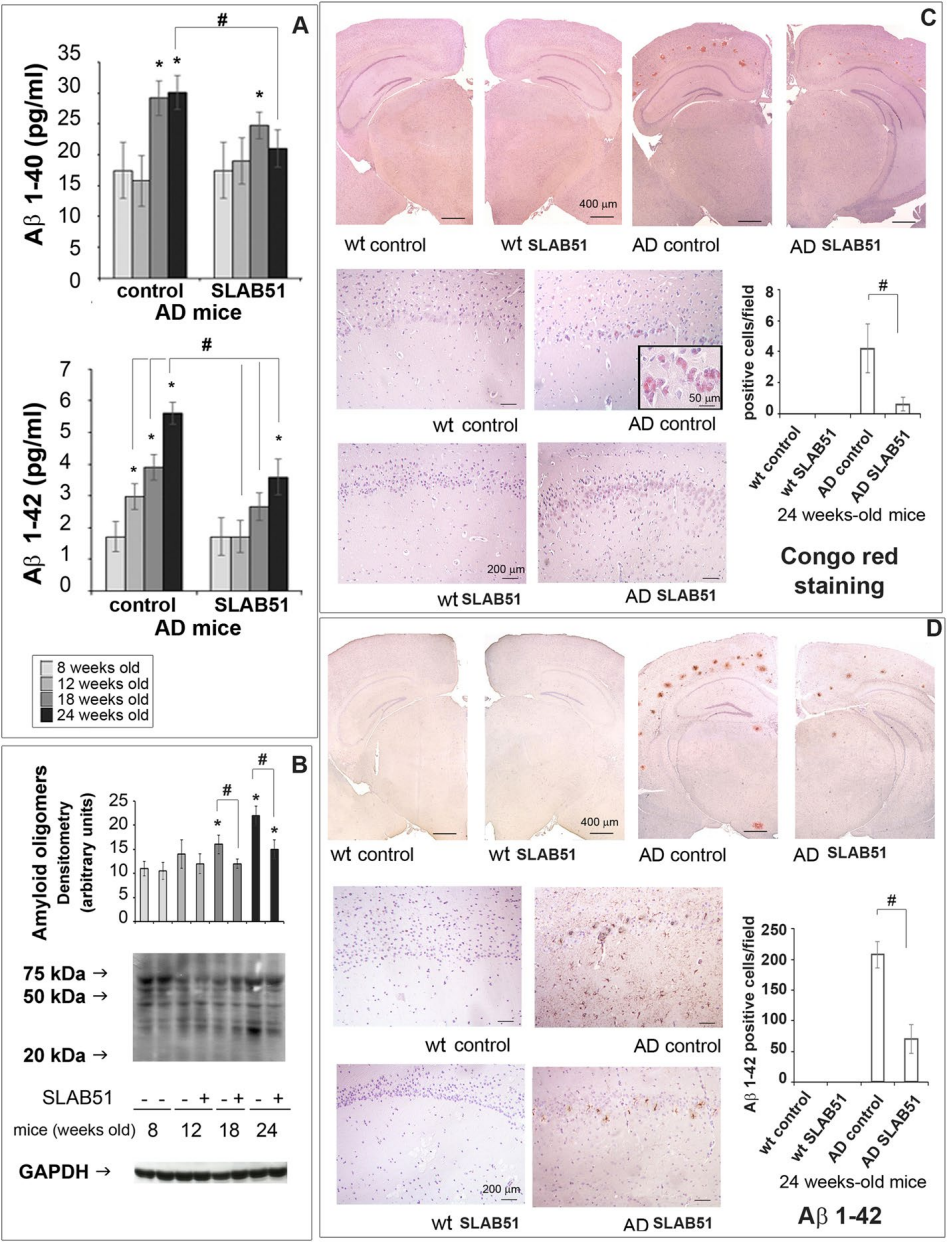
Aβ load. Panel A: Aβ_1–40_ and Aβ_1–42_ levels expressed as pg/ml determined by ELISA in the brains of AD mice treated or not with SLAB51 (n = 8). Panel B: Expression levels of amyloid oligomers detected by western blot. The densitometry from five separate blots and a representative immunoblot are reported. Equal protein loading was verified by using an anti-GAPDH antibody. The detection was executed by ECL. Data points marked with an asterisk are statistically significant compared to 8 weeks-old controls (*p < 0.05). Data points marked with hash are statistically significant compared to their respective control mice in the same time point (^#^p < 0.05). Uncropped gels are reported in Supplemental Figure 1. Panel C: Congo red staining of extra- and intra-cellular amyloid deposits in 24-week-old wt and AD mice administered with water or SLAB51 (groups’ size is 8). Specific Congo red staining was progressively seen in somata and processes of hippocampal Ammon’s horn pyramidal cells (insert), especially in untreated AD mice. Strong extracellular deposits demonstrate the formation of amyloid plaques, also visualized by immunostaining. (Congo red stain, with Meyer’s hematoxylin nuclear counterstain. Coronal sections, Bar = 400 μm; *dentate gyrus* magnification, Bar = 200 μm; insert, Bar = 50 μm). Data are presented as positive cells/field and are representative of 5 histological section for each brain (n = 8 per sub-group). Data points marked with a hash are statistically significant compared to their respective water-treated mice (p < 0.05). Panel D. Aβ_1–42_ IHC stain: wt and AD mice administered with water (control) or SLAB51. In both upper (low magnification) and lower (high magnifications) groups of images, immunoreactivity towards Aβ_1–42_ peptide (Aβ_1–42_ C-terminus pAb, Millipore) was progressively seen in somata and processes of hippocampal pyramidal cells and cortical neurons of AD untreated mice. A strong extracellular reactivity, associated with Aβ plaque formation, can be observed in both treated and untreated AD mice (IHC stain, with Meyer’s hematoxylin nuclear counterstain. Coronal sections, Bar = 400 μm; *dentate gyrus* magnification, Bar = 200 μm). The histogram shows the Aβ_1–42_ positive cells/field. Data represent 5 histological section for each brain (n = 8). Data points marked with a hash are statistically significant compared to their respective water-treated mice (p < 0.05).

To assess if SLAB51 treatment had effects on brain Aβ load, we first measured the levels of Aβ_1–40_ and Aβ_1–42_ peptides. As expected, amyloid peptides did not increase in wild type mice (data not shown), Interestingly, Aβ_1–42_ load was significantly reduced in 12-week-old AD mice treated with SLAB51 compared to controls. No significant effect on Aβ_1–40_ levels was observed (Fig. 5, panel A). We then evaluated the accumulation of amyloid oligomers through western blotting and found a considerable reduction of these toxic structures in treated AD mice, at both 18 weeks and 24 weeks of age, compared to controls (Fig. 5, panel B). Congo red staining of brain Aβ plaques evidenced a significant reduction of extracellular amyloid deposits, associated with substantially lower levels of staining of somata and processes of hippocampal pyramidal cells from Ammon’s horn, and in granule cells from dentate gyrus, especially in sections of AD mice treated with the SLAB51 mixture (Fig. 5, panel C). Regarding the effects of SLAB51 on wt animals, no significant changes in the amount of amyloid peptides were observed, as also demonstrated histologically in Congo red stained sections (Fig. 5, panel C). Moreover, immunoreactivity towards Aβ_1–42_ peptide was progressively seen in somata and processes of hippocampal pyramidal cells and cortical neurons of untreated AD mice. Lower amounts of Aβ_1–42_ deposits were immunodetected in SLAB51 treated AD mice (Fig. 5, panel D).

### Effect of SLAB51 mixture on proteasomal and autophagic proteolytic activity

**Figure 6:**
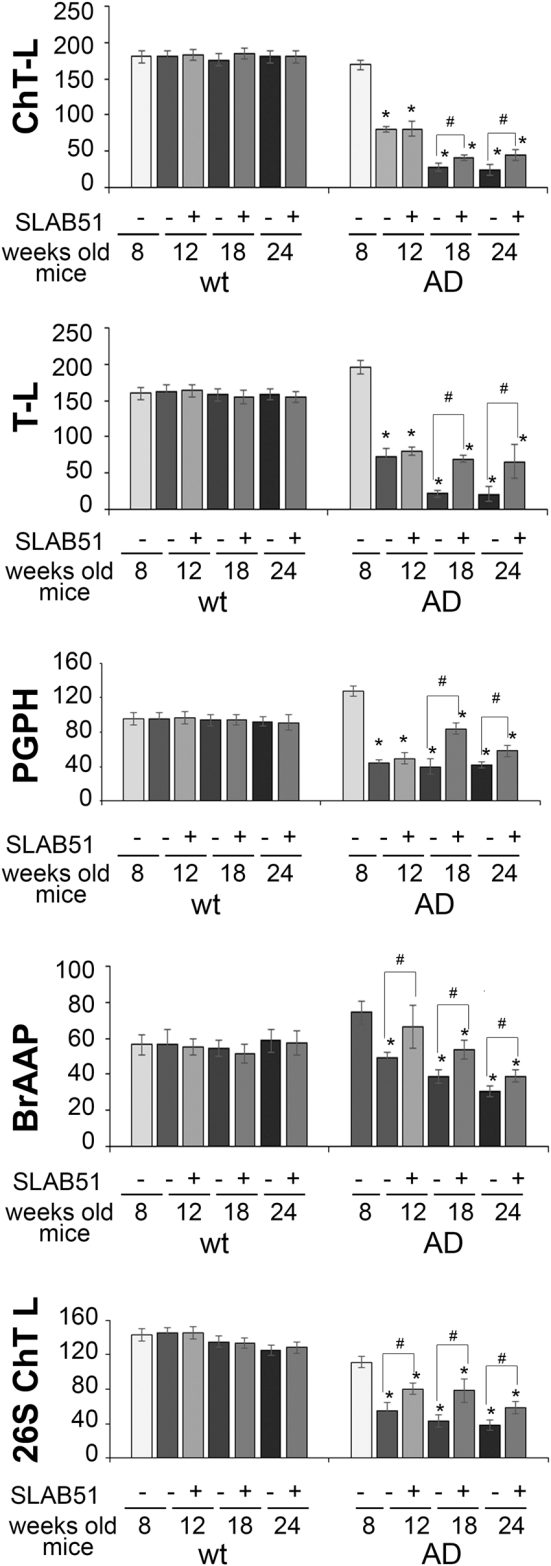
Effect of SLAB51 on proteasomal activity. Proteasome activity in SLAB51 treated and untreated wt (left) and AD (right) mice. The ChT-L, T-L, PGPH and BrAAP activities of the 20S proteasome and the ChT-L activity of the 26S proteasome were measured in brain homogenates as described in the Methods section. Results are expressed as fluorescence units (U. F.). Data points marked with an asterisk are statistically significant compared to untreated 8-week-old mice (*p < 0.05). Data points marked with a hash are statistically significant compared to their respective control mice in the same time point (*p < 0.05).

**Figure 7:**
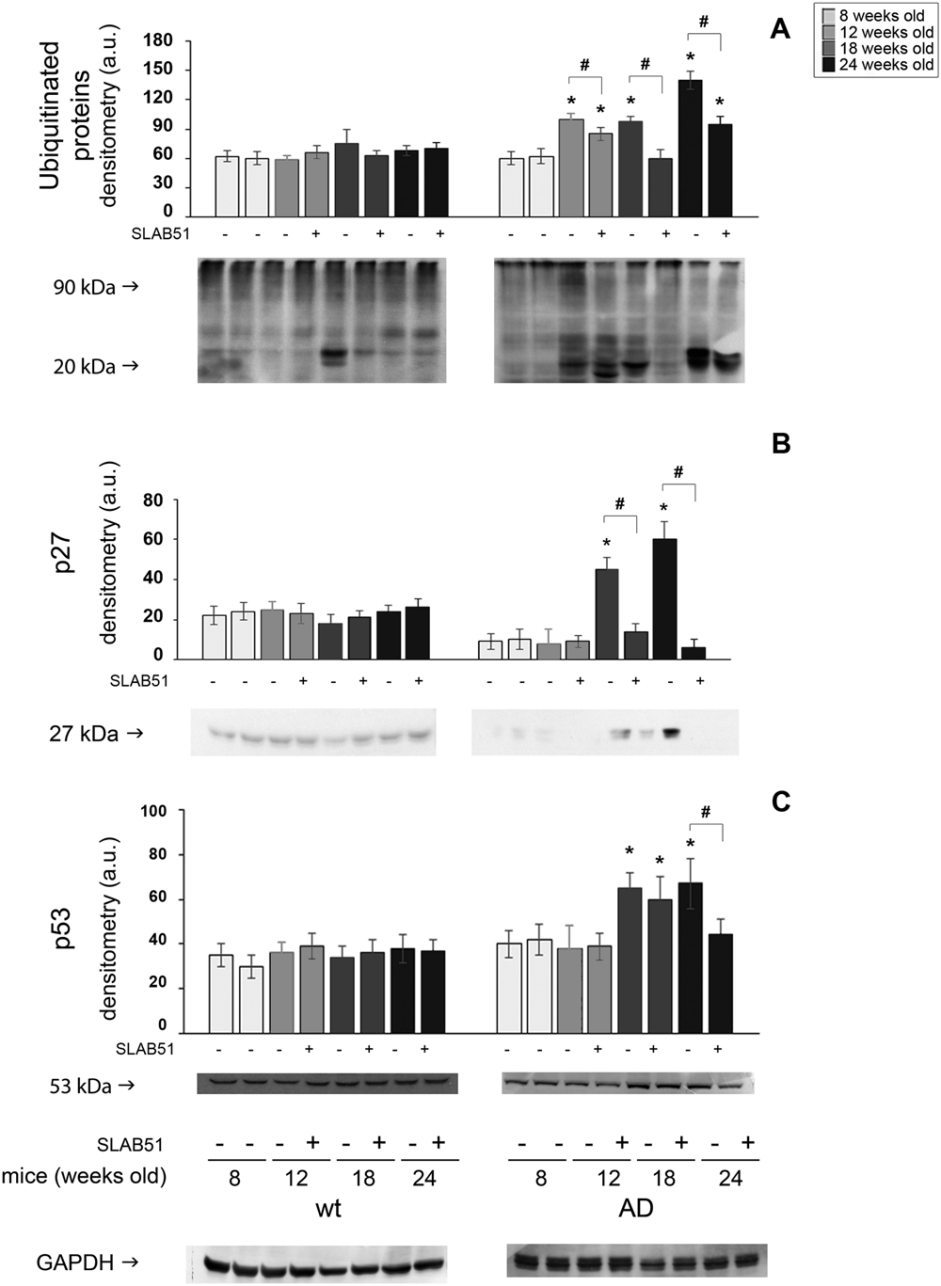
Effect of SLAB51 on proteasomal substrates. Detection of the levels of ubiquitinated proteins (panel A), p27 (panel B) and p53 (panel C) in SLAB51 treated and untreated wt and AD mice. The densitometric analyses obtained from five separate blots and representative immunoblots are shown. Equal protein loading was verified by using an anti-GAPDH antibody. The detection was executed by an ECL western blotting analysis system. Data points marked with an asterisk are statistically significant compared to 8-week-old control mice (*p < 0.05). Data points marked with a hash are statistically significant compared to their respective control mice at the same time point (^#^p < 0.05). Uncropped gels are reported in Supplemental Figure 1.

**Figure 8:**
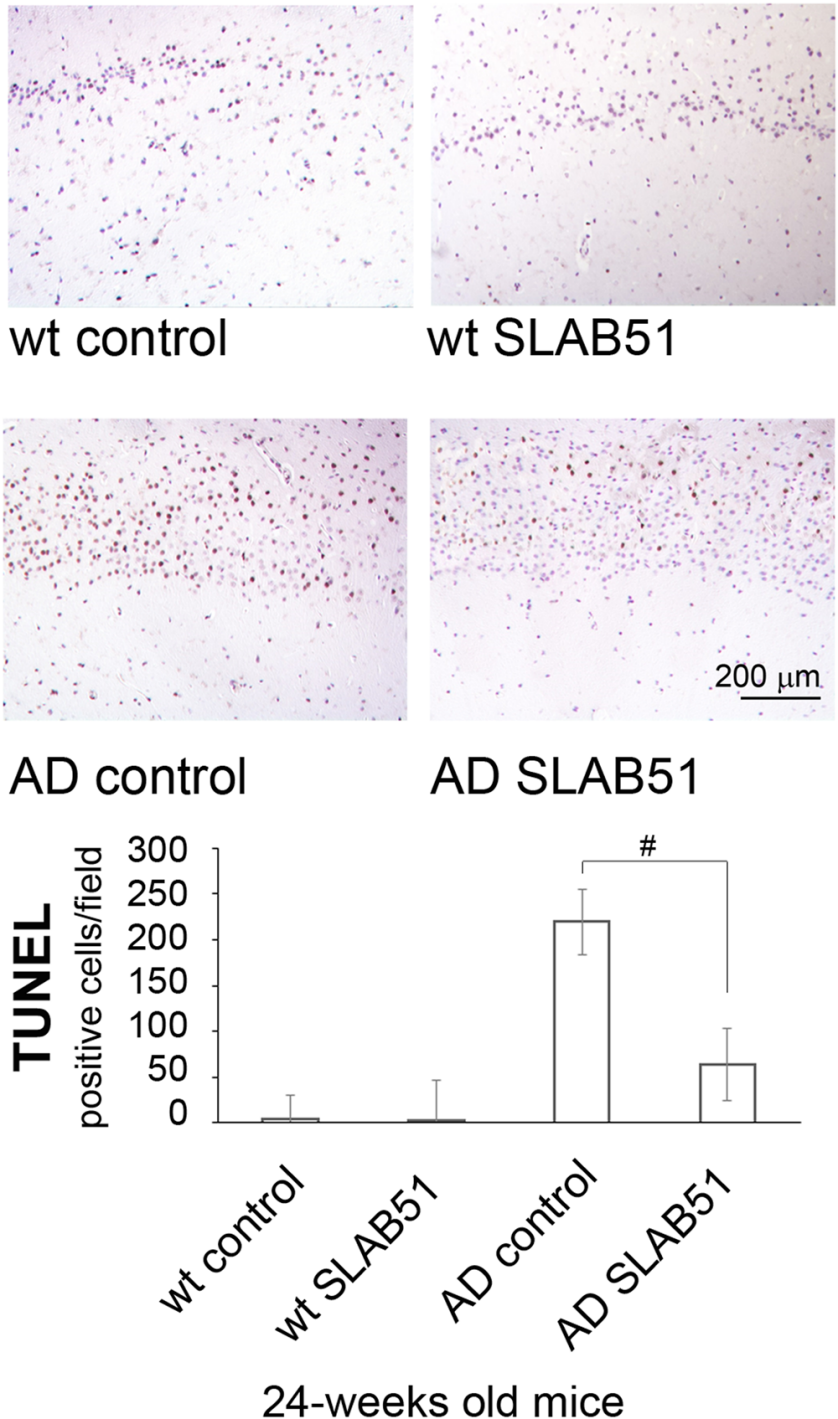
TUNEL detection of apoptotic neurons in hippocampal area of SLAB51 treated and untreated AD and wt mice. Apoptotic cells are characterized by black-brownish nuclear stain, as shown in the representative images. (TUNEL (DeadEnd, Promega®) reaction, with Meyer’s hematoxylin nuclear counterstain. Bar = 200 μm.). The histogram shows the TUNEL positive cells/field. Data are representative of 5 histological section for each brain (n = 8 per sub-group). Data points marked with a hash are statistically significant compared to their respective water-treated mice (p < 0.05).

Brain homogenates were analyzed through enzymatic assays and western blotting analyses in order to monitor the functionality and expression levels of components of the proteolytic pathways UPS and autophagy. In wt mice, SLAB51 did not modify proteasome functionality at any time point. A different result was obtained when analyzing AD mice. In fact, proteasome activity decreased in 3xTg-AD control mice, whereas AD mice treated with SLAB51 showed partially restored activity. In particular, 18- and 24-week-old treated AD mice showed increased ChT-L, T-L, and PGPH activity compared to age-matched untreated controls. A re-establishment of BrAAP activity was already evident at 12 weeks of age (Fig. 6). A similar pattern was obtained measuring the ChT-L activity of the 26S proteasome, the complex in charge of the removal of ubiquitinated proteins, where a significant recovery of activity was evident in treated AD mice at each time point (Fig. 6). To confirm these data, we performed western blotting assays to detect p27, p53 and ubiquitinated proteins, known substrates of the proteasome and markers of its functionality. At the analyzed time points, treated and untreated wt mice showed no significant differences in the expression levels of these proteins (Fig. 7). As expected, AD mice displayed a considerable accumulation of the analyzed substrates due to the strong proteasome impairment typical of neurodegeneration^61^. Figure 6 indicates that in AD mice, SLAB51 treatment induced a decrease in the levels of these markers in agreement with the data from the activity assays. The reduction in the buildup of ubiquitinated proteins induced by SLAB51 was significant in 18-week-old mice, whereas the effects on p27 and p53 were already evident in 12-week-old mice. The accumulation of these substrates, in particular p53, in treated and control AD mice, correlates with the significantly different apoptotic index at the neuronal level, especially in the granule cells layer from the hippocampal area of dentate gyrus (Fig. 8), and also in the pyramidal cells layer from Ammon’s horn of the hippocampal area. No statistically significant differences in apoptotic levels were observed in 24-week-old treated AD mice and in treated or control wt mice. The considerable accumulation of p53 due to the strong proteasome impairment observed in untreated AD mice confirms the ability of this substrate to trigger the apoptotic pathway; as expected, treatment with the probiotic mixture induced a considerable decrease in apoptotic activity, regulating the levels of proteasome substrates.

**Figure 9:**
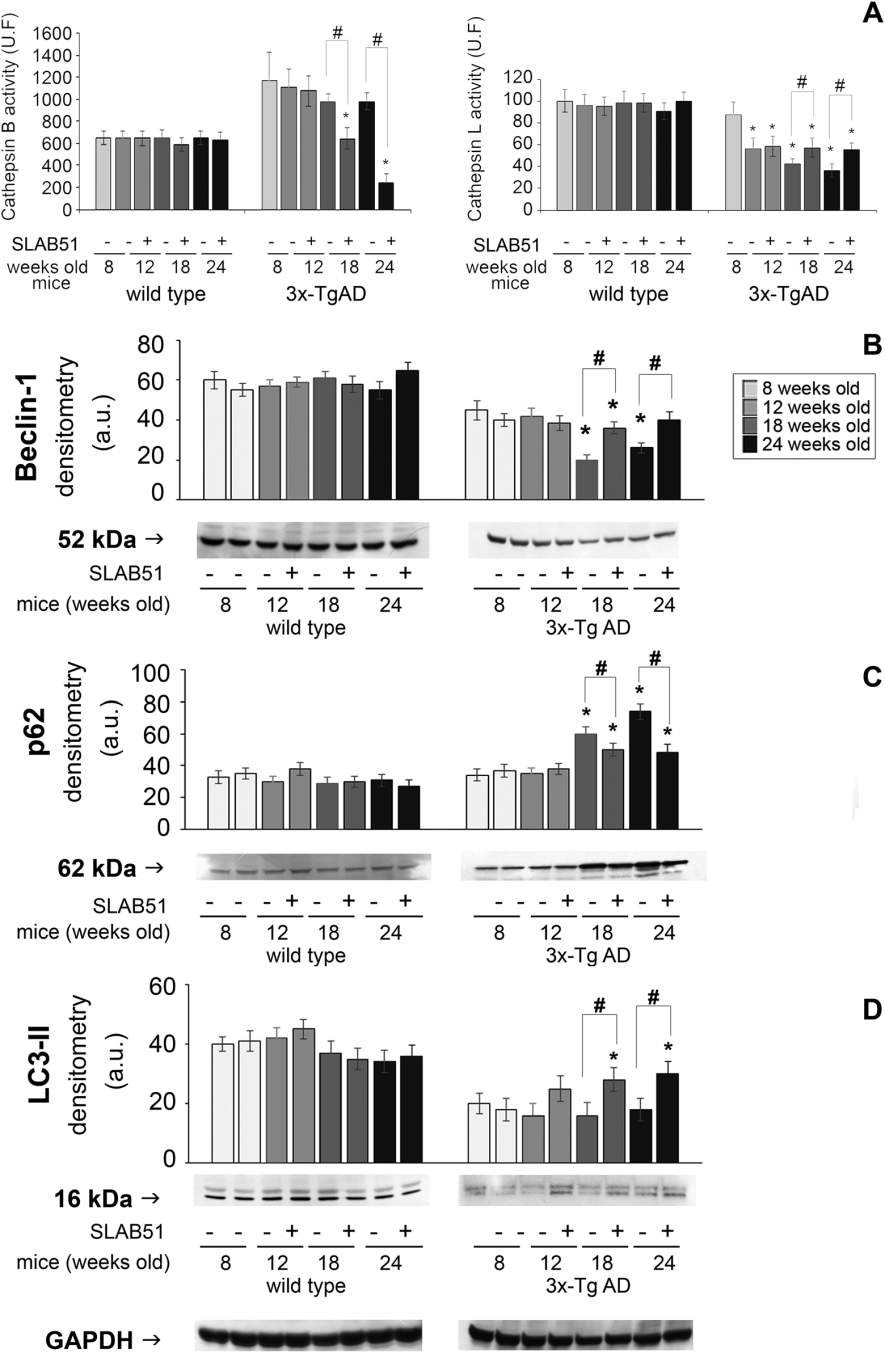
Autophagic markers. Panel A: Cathepsin B and cathepsin L activity in SLAB51 treated and untreated wt and AD mice. Results are expressed as fluorescence units. Data points marked with an asterisk are statistically significant compared to their respective untreated control mice (*p < 0.05). Panels B,C,D: levels of the autophagy-related proteins Beclin 1, p62 and LC3-II in SLAB51 treated and untreated wt and AD mice. Representative immunoblots and corresponding densitometric analyses obtained from five separate blots are shown. Equal protein loading was verified by using an anti-GAPDH antibody. The detection was executed by an ECL western blotting analysis system. Data points marked with an asterisk are statistically significant compared to 8-week-old control mice (*p < 0.05). Data points marked with a hash are statistically significant compared to their respective control mice at the same time point (^#^p < 0.05) Original membranes strips are reported in Supplemental Figure 1.

Among the lysosomal enzymes, cathepsin B and cathepsin L were evaluated. Cathepsin B (CatB) is a cysteine protease associated with amyloid plaques and suggested to reduce Aβ levels^62^. A decrease in Cathepsin B activity was observed in 18- and 24-week-old treated mice compared with controls. Conversely, SLAB51 was able to restore cathepsin L (CatL) activity in 18- and 24-week-old mice compared with controls (Fig. 9). This finding is of particular interest considering the ability of the enzyme to increase α-secretase activity, thereby suppressing Aβ levels^63^. Exposure to SLAB51 did not change the activity of either enzyme in wt mice.

The levels of the autophagy-related proteins beclin-1, LC3-II, and p62 were detected through western blotting assays. Beclin-1 plays a key role in autophagy, being involved in the enrolment of membranes to form autophagosomes^64^. LC3-II is tightly bound to the autophagosomal membranes and is an established autophagic marker^65^. p62 binds to both LC3-II and ubiquitin, and is finally degraded in autophagolysosomes. Therefore, p62 levels inversely correlate with autophagic activity^66^. Our data show that in AD mice, treatment with SLAB51 increased the levels of beclin-1 and LC3-II and decreased the amounts of p62, suggesting an activation of the autophagic flux (Fig. 9).

## Discussion

AD is a progressive neurodegenerative disorder, with age the major risk factor. AD patients are characterized by cognitive impairment and dementia, the accumulation of neurofibrillary tangles and Aβ senile plaques, neurite and brain cell atrophy, and increased oxidative stress and pro-inflammatory signals^67, 68^. Interestingly, the role of microbes in both aging and the onset and progression of AD has been emerging in recent years^26, 69^. In this regard, studies conducted on animal models showed that modifications of gut microbiota induced by oral bacteriotherapy reflect changes in genes involved in inflammatory and neuronal plasticity processes, with a positive impact on neuronal function^33, 70^.

In the present work, we assessed the potential beneficial effects of modulating gut microbiota composition by a four-month treatment with SLAB51 (a mixture of lactic acid bacteria and bifidobacteria) in a triple transgenic mouse model of AD, 3x-Tg AD mice^38^, in the early stages of the disease.

Significant differences in microbiota structure were observed at all time points between the wt and the 3xTg-AD mice, and these differences were independent of treatment. However, treatment with SLAB51 induced larger shifts in microbial communities in the 3xTg-AD mice, as evidenced by more pronounced changes in Unifrac distances. Interestingly, this did not lead to obvious differences in abundances of specific bacterial taxa, suggesting more gradual shifts across the entire microbiota. However, the functional content as predicted by PICRUSt was associated with more changes due to SLAB51 in AD mice, as more pathways were increased due to treatment. Of interest is that SLAB51 induced several metabolic pathways associated with energy metabolism, amino acid metabolism, and nucleotide metabolism.

The increase in *Bifidobacterium spp*. and the reduction in *Campylobacterales* observed in AD mice upon administration with SLAB51 is important for the role of these bacteria in inflammatory pathways. In fact, *Bifidobacterium* strains possess anti-inflammatory properties principally attributed to small heat-stable, non-lipophilic compounds resistant to protease and nuclease treatments^71^. Moreover, certain species of genus *Bifidobacterium* could negatively modulate mRNA levels of pro-inflammatory cytokines produced from LPS-stimulated macrophages^72^. Instead, immune-stimulatory effects of *Campylobacter jejuni* and *Campylobacter coli* have been observed on peripheral blood mononuclear cells^73^. Additionally, *in vivo* studies demonstrated that the purified lipooligosaccharid of *Campylobacter jejuni* increased the expression of pro-inflammatory cytokines in chickens^74^. Upon SLAB51 administration reduced plasma concentrations of pro-inflammatory cytokines have been observed in AD mice (Fig. 3), confirming that modification of microbiota produced anti-inflammatory effects in probiotic administered subjects. Interestingly treated AD mice possess higher levels of G-CSF that is a modulator of systemic immune responses by inhibiting pro-inflammatory cytokines and has been demonstrated to decrease β-amyloid deposition and to reverse cognitive impairment in an AD mice model^60^.

Behavioral tests highlight a positive effect of oral treatment with SLAB51 on behavioral performance in AD mice, suggesting the restoration of hippocampus functions, in agreement with published evidence supporting the idea that cognitive functions are affected by bacteria acting through the gut-brain axis. Some authors have demonstrated that certain strains of *Lactobacillus* and *Bifidobacterium* secrete essential neurotransmitters such as gamma-aminobutyric acid (GABA) and acetylcholine that mediate the positive impact of probiotics on behavior in neurological dysfunctions^75^. Additionally, an impact on anxiety of *Campylobacter jejuni* infection has been previously reported in central nervous system disorders^76^. In our study, the improved cognitive activity in treated AD mice did not correlate with different levels of anxiety. Interestingly, analysis of microbiota showed a reduced presence of *Campylobacterales* in the treated AD group compared with the untreated AD group (Supplementary Table 2). This result is valuable also considering that a high prevalence of *Campylobacterales* infections have been observed in AD patients and, after *Helicobacter pylori* eradication, cognitive and functional status parameters ameliorated^77^.

Improvement of cognitive function is supported by increased plasma concentration of gut hormones such as ghrelin, leptin, GLP1 and GIP. Peptide hormones secreted in the gut play a role in modulating nervous functions like learning and memory. The time-dependent decrease of both ghrelin and leptin plasma concentrations in AD mice are in agreement with previously published data showing altered peripheral levels of these hormones in AD patients^8, 78^. Moreover, some authors previously demonstrated 3–4 months-old male 3xTg-AD mice exhibit significantly higher basal mineralocorticoids and glucocorticoids mRNA levels in the hippocampus and increased glucocorticoids but decreased corticotrophin releasing hormone mRNA levels in the paraventricular nucleus of the hypothalamus compared to male WT mice^79^. Thus, the progressive decrease of circulating metabolic hormones concentration in untreated AD mice may be due to the unbalanced hypothalamic-pituitary-adrenal axis. In fact, these hormones are affected by several factors such as stress, and glucocorticoids levels^80^. Interestingly, mice treated with the probiotic mixture showed higher plasma levels of such hormones, and this is important because ghrelin has been proven to counteract memory deficits and synaptic degeneration in AD animal models^9^, and leptin has been demonstrated to act as neurotrophic factor and to exert neuroprotective effects against toxicity induced by Aβ oligomers *in vitro* ^5, 6^. These data correlate with the decreased Aβ deposits in the brains of treated AD mice, and they are in good agreement with evidence showing that plasma leptin concentration is negatively associated to Aβ levels due to a direct regulatory effect on the γ-secretase-mediated amyloidogenic pathway^10^. The age-related decline of plasma ghrelin concentration and the impairment of ghrelin signaling observed in AD patients are closely related to the compromised memory and learning processes. Oral administration of SLAB51 enhanced GLP-1 and GIP plasma concentrations in AD mice, resulting in a neuroprotective effect of both incretins^81^. Again, GLP-1 has been shown to reduce Aβ load *in vivo* and in cultured neuronal cells^14^. Interestingly, following SLAB51 administration an increase of the bacterial metabolites SCFAs has been detected in feces of AD mice. Considering that bacterial by products such as SCFAs exert a number of neuromodulator effects and directly act on gastrointestinal cells stimulating the synthesis of hormones like leptin and GLP-1^34^, our data contribute to define the link between gut microbiota modulation and metabolic hormones. Moreover, the enriched gut concentration of SCFAs, well recognized anti-inflammatory bioactive metabolites^82, 83^ is encouraging also because of the number of evidences supporting SCFAs therapeutic potential in neurodegenerations^84^. For example, histone deacetylase inhibitor 4-phenylbutyrate is responsible for restoring fear learning, counteracting intraneuronal Aβ deposition, and regulating dendritic spine density by exerting a chaperone-like activity and via the transcriptional activation of key proteins in synaptic plasticity and structural remodeling^85^; Here we show that SLAB51 administration exerts multiple effects by modulating gut microbiota composition and causing metabolic changes, such as the increase of SCFAs able to directly act in the gut and in the brain, due to their ability to pass the blood brain barrier^86^.

Innovatively, we show that probiotics counteracted the typical morphological alterations of AD, including the reduction in brain weight, the decline of cortical areas, and the general brain damage and shrinkage. Furthermore, SLAB51 contributed to a consistent reduction in the amount of cerebral Aβ, both in the form of peptides and oligomers. Consequently, a decreased number and size of amyloid plaques were observed upon treatment. Considering a recently published work in which *Lactobacillus helveticus* ameliorated APP metabolism in cell-based assays, favored memory in mice, and reduced Aβ_1–40_ serum concentration in rats^87^, our findings prove a beneficial role for probiotic bacteria in AD subjects, identifying a synergistic effect obtained through the use of a successful formulation.

Exploring proteolytic pathways, normally impaired in AD, we evaluated the ability of the treatment to restore their functionality in neurons. Several papers have described dramatically impaired proteasome functionality in AD^1, 88^. For the first time, the probiotic mixture partially restored both 20S and 26S proteasomes activities. Enzymatic activities were confirmed through western blotting analyses investigating the levels of specific proteasomal substrates, such as ubiquitin conjugates, p27 and p53 proteins. All these proteins accumulated with proteasome inhibition, whereas their levels decreased upon SLAB51 treatment. In particular, as the treatment restores proteasome functionality, p53 levels diminished and a decrease in apoptotic levels was detected in granule cells from dentate gyrus of hippocampal areas, and in some pyramidal cells of the Ammon’s horn of AD treated mice. An increase in p53 immunoreactivity has been previously observed in sporadic AD^89, 90^, especially in cortical neurons undergoing neurofibrillary degeneration^91^. The time-dependent p53 increase in untreated AD mice is also in agreement with a work demonstrating that p53 is upregulated approximately 2-fold in the superior temporal gyrus of Alzheimer’s patients compared to healthy elderly control subjects^92^.

Moreover, SLAB51 administration triggered autophagy, as demonstrated by the decrease of cathepsin B activity and the increase of cathepsin L activity. These lysosomal enzymes are differently involved in AD development: the cysteine protease cathepsin B is associated with amyloid plaques in AD brains and has been suggested to be responsible for the increase in Aβ production. Conversely, cathepsin L activity increases α-secretase-mediated non-amyloidogenic pathway. Western blotting of autophagy markers confirmed that this pathway is activated in treated AD mice. Therefore another pioneering piece of evidence is that the SLAB51 probiotic formulation restored altered autophagy in AD. Collectively, biochemical analyses on proteolysis demonstrate that SLAB51 probiotic mixture significantly influences proteolysis, restoring impaired proteasome activities and consequently modulating the autophagic flux, confirming the crosstalk between the two pathways^1^. We believe that SLAB51’s positive effects on proteolysis are mediated by increased gut hormones. In fact, in a recent paper we demonstrated that ghrelin controls neural homeostasis through direct regulation of UPS and autophagy in AD neuronal cells^93^.

Furthermore, it has been demonstrated that autophagy negatively regulates Wingless-related integration site (Wnt) signaling, which is involved in cell migration, apoptosis and differentiation of the CNS^94^, and its targets, among them FGF9. Our results show that treatment with SLAB51 restores autophagy and modulates gut hormone production in AD mice. These results suggest that microbiota composition influences Wnt signaling and provide an explanation of the reduced expression of FGF9 in the CNS. Increased levels of FGF9 in the hippocampus of subjects with major depressive disorder have been observed. In rodent models of affective disorders, chronic social defeat stress increased anxiety, compromised social interaction, and decreased body weight, and was associated with increased hippocampal FGF9 expression^95^. Accordingly, FGF-9 immunoreactivity has been detected in human hippocampal and cortical neurons, astrocytes, and dystrophic neurites of senile plaques, in hippocampal sections of AD patients^96^. In this study an association between hippocampal FGF9 expression and severity of lesions related to AD in mice was observed. Therefore, reduced hippocampal expression of FGF9 in 24-week-old AD mice treated with SLAB51, comparable to wt mice FGF9 hippocampal expression, contributes to the improvement of behavioral performances.

Previous studies demonstrated that modifications to the microbiota modulate the expression of several genes and regulate neurotransmitter and synaptic-related proteins levels, thus influencing brain development and function^33, 97^.

## Conclusions

In this paper we provide evidence that by modulating gut microbiota several pathways are affected, consequently delaying AD progression. In 3xTg-AD mice, SLAB51 changed the composition of gut microbiota and its metabolites, positively interfering with inflammatory cytokines, gut hormones concentration and proteolysis, reducing Aβ load and improving cognitive function, demonstrating a role in the prevention and treatment of AD and supporting the idea of the gut microbiota modulation to counteract Aβ-mediated AD-type pathogenic processes.

## Electronic supplementary material

Supplemental information
