# Supplementary material for: Microbiota modulation counteracts Alzheimer’s disease progression influencing neuronal proteolysis and gut hormones plasma levels
Source: Sci Rep. 2017 May 25;7:2426. doi: 10.1038/s41598-017-02587-2 (PMC5445077; doi:10.1038/s41598-017-02587-2)

Supplemental information:

TITLE: Microbiota modulation counteracts Alzheimer's disease progression influencing neuronal proteolysis and gut hormones plasma levels

AUTHORS: Bonfili Laura[*1], Cecarini Valentina[1], Berardi Sara[1], Scarpona Silvia[1], Suchodolski Jan S.[2], Nasuti Cinzia[3], Fiorini Dennis [4], Boarelli Maria Chiara [4], Rossi Giacomo[1], Eleuteri Anna Maria[1]

[1] School of Biosciences and Veterinary Medicine, University of Camerino, via Gentile III da Varano, 62032 Camerino (MC), ITALY

[2] Gastrointestinal Laboratory, Department of Small Animal Clinical Sciences, College of Veterinary Medicine and Biomedical Sciences, Texas A&M University, USA

[3] School of Pharmacy, Pharmacology Unit, University of Camerino, via Madonna delle Carceri, 62032 Camerino (MC), ITALY

[4] Division of Chemistry, School of Science and Technology, University of Camerino, I-62032 Camerino, MC, Italy.

Supplemental Table S1: Differences in bacterial taxa between 24 weeks-old wt mice and 3xTg-AD mice (untreated).

	Alzheimer Control		Wild Type Control			
**Taxa**	**mean**	**SD**	**mean**	**SD**	**p-value**	**q-value**
**Phylum**						
Unassigned;other	0.15	0.03	0.19	0.03	0.048	0.119
Actinobacteria	0.10	0.07	0.06	0.02	0.248	0.276
Bacteroidetes	41.78	16.82	51.20	7.27	0.149	0.248
Cyanobacteria	0.01	0.00	0.05	0.03	0.001	0.003
Deferribacteres	0.00	0.00	0.00	0.00	0.246	0.276
Firmicutes	42.99	15.25	35.58	5.91	0.248	0.276
Proteobacteria	14.52	11.52	8.07	3.62	0.336	0.336
TM7	0.05	0.05	0.16	0.10	0.024	0.079
Tenericutes	0.37	0.25	2.13	0.60	0.001	0.003
Verrucomicrobia	0.03	0.02	2.56	4.63	0.083	0.165
**Class**						
Unassigned;Other	0.15	0.03	0.19	0.03	0.048	0.172
Actinobacteria	0.00	0.00	0.00	0.00	0.916	0.916
Coriobacteriia	0.10	0.07	0.06	0.01	0.268	0.424
Bacteroidia	41.77	16.82	51.19	7.27	0.149	0.314
phingobacteriia	0.00	0.01	0.02	0.02	0.143	0.314
4C0d-2	0.00	0.00	0.04	0.03	0.001	0.005

Chloroplast	0.01	0.00	0.01	0.00	0.728	0.777
Deferribacteres	0.00	0.00	0.00	0.00	0.246	0.424
Bacilli	3.63	5.57	2.67	1.23	0.248	0.424
Clostridia	39.16	14.36	32.82	5.78	0.501	0.594
Erysipelotrichi	0.20	0.14	0.10	0.04	0.470	0.594
Alphaproteobacteria	0.27	0.67	0.21	0.17	0.034	0.162
Betaproteobacteria	0.14	0.24	0.07	0.04	0.736	0.777
Deltaproteobacteria	0.48	0.39	0.19	0.16	0.054	0.172
Epsilonproteobacteria	13.60	11.89	7.57	3.53	0.441	0.594
Gammaproteobacteria	0.02	0.02	0.03	0.01	0.439	0.594
TM7-3	0.05	0.05	0.16	0.10	0.024	0.149
Mollicutes	0.37	0.25	2.13	0.60	0.001	0.005
Verrucomicrobiae	0.03	0.02	2.56	4.63	0.083	0.224
**Order**						
Unassigned;other	0.15	0.03	0.19	0.03	0.048	0.167
Bifidobacteriales	0.00	0.00	0.00	0.00	0.584	0.654
Coriobacteriales	0.10	0.07	0.06	0.01	0.268	0.441
Bacteroidales	41.77	16.82	51.19	7.27	0.149	0.321
Sphingobacteriales	0.00	0.01	0.02	0.02	0.143	0.321
YS2	0.00	0.00	0.04	0.03	0.000	0.006
Streptophyta	0.01	0.00	0.00	0.01	0.548	0.639
Deferribacterales	0.00	0.00	0.00	0.00	0.246	0.430
Bacillales	0.00	0.01	0.00	0.00	0.101	0.257
Lactobacillales	2.47	2.65	2.64	1.23	0.211	0.422
Turicibacterales	1.15	3.13	0.02	0.01	0.033	0.130
Clostridiales	39.16	14.36	32.82	5.78	0.501	0.637
Erysipelotrichales	0.20	0.14	0.10	0.04	0.470	0.627
RF32	0.00	0.00	0.14	0.14	0.000	0.006
Rhizobiales	0.23	0.63	0.03	0.05	0.923	0.923
Rickettsiales	0.03	0.04	0.02	0.03	0.626	0.674
Sphingomonadales	0.00	0.00	0.01	0.01	0.403	0.594
Burkholderiales	0.14	0.24	0.07	0.04	0.700	0.726
Desulfovibrionales	0.48	0.39	0.19	0.16	0.054	0.168
Campylobacterales	13.60	11.89	7.57	3.53	0.441	0.618
Enterobacteriales	0.01	0.01	0.02	0.01	0.245	0.430
Pseudomonadales	0.00	0.00	0.00	0.00	0.290	0.451
Xanthomonadales	0.00	0.01	0.00	0.00	0.547	0.639
CW040	0.05	0.05	0.16	0.10	0.024	0.110
Anaeroplasmatales	0.18	0.22	1.32	0.64	0.001	0.010
Mycoplasmatales	0.01	0.01	0.40	0.51	0.015	0.083
RF39	0.18	0.15	0.41	0.17	0.005	0.037
Verrucomicrobiales	0.03	0.02	2.56	4.63	0.083	0.232
**Family**						
Unassigned;Other	0.15	0.03	0.19	0.03	0.048	0.156
Bifidobacteriaceae	0.00	0.00	0.00	0.00	0.584	0.656
Coriobacteriaceae	0.10	0.07	0.06	0.01	0.268	0.456
o__Bacteroidales;Other	0.00	0.00	0.00	0.00	0.155	0.355
;o__Bacteroidales;f__	0.09	0.09	0.01	0.01	0.021	0.086
Bacteroidaceae	2.41	3.09	4.04	1.69	0.068	0.183
Prevotellaceae	0.01	0.01	0.07	0.10	0.698	0.731
Rikenellaceae	3.88	2.51	4.33	1.91	0.564	0.648
S24-7	28.23	15.35	37.13	6.31	0.248	0.439

[Odoribacteraceae]	4.45	4.34	5.59	2.07	0.211	0.439
[Paraprevotellaceae]	2.69	3.03	0.01	0.01	0.001	0.008
Sphingobacteriaceae	0.00	0.01	0.02	0.02	0.143	0.346
o__YS2;f__	0.00	0.00	0.04	0.03	0.000	0.008
o__Streptophyta;f__	0.01	0.00	0.00	0.01	0.548	0.648
Deferribacteraceae	0.00	0.00	0.00	0.00	0.246	0.439
Enterococcaceae	0.04	0.10	0.00	0.00	0.002	0.017
Lactobacillaceae	2.40	2.66	2.64	1.23	0.178	0.390
Streptococcaceae	0.03	0.02	0.01	0.00	0.011	0.063
Turicibacteraceae	1.15	3.13	0.02	0.01	0.033	0.115
o__Clostridiales;Other	0.68	1.46	0.04	0.02	0.434	0.580
o__Clostridiales;f__	22.93	7.48	19.72	3.24	0.441	0.580
Christensenellaceae	0.01	0.01	0.00	0.00	0.055	0.158
Clostridiaceae	0.31	0.29	0.13	0.08	0.387	0.574
Dehalobacteriaceae	0.18	0.08	0.23	0.07	0.229	0.439
Lachnospiraceae	9.56	6.29	7.64	4.16	0.387	0.574
Peptococcaceae	0.04	0.05	0.02	0.01	0.885	0.904
Peptostreptococcaceae	0.03	0.06	0.00	0.00	0.018	0.081
Ruminococcaceae	5.37	1.65	5.02	1.12	0.501	0.622
[Mogibacteriaceae]	0.06	0.04	0.01	0.01	0.002	0.016
Erysipelotrichaceae	0.20	0.14	0.10	0.04	0.470	0.601
o__RF32;f__	0.00	0.00	0.14	0.14	0.000	0.008
Methylobacteriaceae	0.00	0.00	0.00	0.00	0.327	0.537
Phyllobacteriaceae	0.23	0.63	0.03	0.05	0.919	0.919
Rhizobiaceae	0.00	0.01	0.00	0.01	0.550	0.648
mitochondria	0.03	0.04	0.02	0.03	0.698	0.731
Sphingomonadaceae	0.00	0.00	0.01	0.01	0.403	0.579
Alcaligenaceae	0.13	0.24	0.05	0.03	0.699	0.731
Oxalobacteraceae	0.01	0.01	0.01	0.01	0.387	0.574
Desulfovibrionaceae	0.48	0.39	0.19	0.16	0.054	0.158
Helicobacteraceae	13.60	11.89	7.57	3.53	0.441	0.580
Enterobacteriaceae	0.01	0.01	0.02	0.01	0.245	0.439
F16	0.05	0.05	0.16	0.10	0.024	0.090
Anaeroplasmataceae	0.18	0.22	1.32	0.64	0.001	0.013
Mycoplasmataceae	0.01	0.01	0.40	0.51	0.015	0.076
o__RF39;f__	0.18	0.15	0.41	0.17	0.005	0.035
Verrucomicrobiaceae	0.03	0.02	2.56	4.63	0.083	0.211
Genus						
Unassigned;Other;Other;Other;Other;Other	0.15	0.03	0.19	0.03	0.048	0.129
Bifidobacterium	0.00	0.00	0.00	0.00	0.584	0.667
Coriobacteriaceae;g__	0.00	0.01	0.00	0.01	0.680	0.755
Adlercreutzia	0.09	0.07	0.05	0.01	0.247	0.367
o__Bacteroidales;Other;Other	0.00	0.00	0.00	0.00	0.155	0.318
o__Bacteroidales;f__;g__	0.09	0.09	0.01	0.01	0.021	0.070
Bacteroides	2.41	3.09	4.04	1.69	0.068	0.170
f__Prevotellaceae;g__	0.01	0.01	0.00	0.00	0.007	0.033
Prevotella	0.00	0.00	0.07	0.10	0.166	0.318
f__Rikenellaceae;g__	3.67	2.38	4.25	1.91	0.564	0.661
AF12	0.09	0.21	0.07	0.06	0.121	0.265
Rikenella	0.12	0.17	0.01	0.01	0.589	0.667
;f__S24-7;g__	28.23	15.35	37.13	6.31	0.248	0.367
Odoribacter	4.45	4.34	5.59	2.07	0.211	0.359
[Prevotella]	2.69	3.03	0.01	0.01	0.001	0.006

Pedobacter	0.00	0.01	0.01	0.02	0.159	0.318
o__YS2;f__;g__	0.00	0.00	0.04	0.03	0.000	0.006
o__Streptophyta;f__;g__	0.01	0.00	0.00	0.01	0.548	0.661
Mucispirillum	0.00	0.00	0.00	0.00	0.246	0.367
Enterococcus	0.04	0.10	0.00	0.00	0.002	0.015
Lactobacillus	2.40	2.66	2.64	1.23	0.178	0.318
Streptococcus	0.03	0.02	0.01	0.00	0.011	0.049
Turicibacter	1.15	3.13	0.02	0.01	0.033	0.100
o__Clostridiales;Other;Other	0.68	1.46	0.04	0.02	0.434	0.577
o__Clostridiales;f__;g__	22.93	7.48	19.72	3.24	0.441	0.577
f__Christensenellaceae;g__	0.01	0.01	0.00	0.00	0.017	0.066
f__Clostridiaceae;g__	0.09	0.08	0.04	0.02	0.162	0.318
Candidatus Arthromitus	0.10	0.13	0.01	0.01	0.091	0.208
Clostridium	0.11	0.16	0.08	0.07	0.441	0.577
SMB53	0.00	0.01	0.00	0.00	0.038	0.109
Dehalobacterium	0.18	0.08	0.23	0.07	0.229	0.367
f__Lachnospiraceae;Other	0.23	0.18	0.13	0.05	0.564	0.661
f__Lachnospiraceae;g__	7.56	5.75	6.30	4.08	1.000	1.000
Anaerostipes	0.01	0.01	0.16	0.09	0.001	0.006
Coprococcus	0.52	0.29	0.53	0.21	0.847	0.900
Dorea	0.09	0.04	0.02	0.01	0.002	0.015
Roseburia	0.00	0.00	0.10	0.09	0.001	0.006
[Ruminococcus]	1.15	0.56	0.39	0.10	0.004	0.022
f__Peptococcaceae;g__	0.04	0.05	0.02	0.01	0.885	0.926
f__Peptostreptococcaceae;g__	0.03	0.06	0.00	0.00	0.018	0.066
f__Ruminococcaceae;Other	0.01	0.01	0.01	0.01	0.261	0.378
f__Ruminococcaceae;g__	1.76	0.49	1.42	0.21	0.178	0.318
g__Oscillospira	2.13	0.76	2.36	0.46	0.564	0.661
;g__Ruminococcus	1.46	0.79	1.23	0.95	0.441	0.577
f__[Mogibacteriaceae];g__	0.06	0.04	0.01	0.01	0.002	0.013
f__Erysipelotrichaceae;g__	0.11	0.06	0.08	0.04	0.563	0.661
Allobaculum	0.06	0.07	0.01	0.01	0.243	0.367
Coprobacillus	0.04	0.05	0.01	0.00	0.056	0.146
o__RF32;f__;g__	0.00	0.00	0.14	0.14	0.000	0.006
Phyllobacterium	0.23	0.63	0.03	0.05	0.919	0.947
f__mitochondria;Other	0.03	0.04	0.02	0.03	0.698	0.755
Sphingomonas	0.00	0.00	0.00	0.00	0.957	0.971
Sutterella	0.13	0.24	0.05	0.03	0.699	0.755
f__Oxalobacteraceae;Other	0.00	0.00	0.01	0.01	0.208	0.359
Desulfovibrionaceae;Other	0.34	0.31	0.02	0.01	0.001	0.006
f__Desulfovibrionaceae;g__	0.00	0.00	0.03	0.03	0.019	0.066
Bilophila	0.01	0.01	0.00	0.00	0.168	0.318
Desulfovibrio	0.13	0.25	0.14	0.15	0.092	0.208
f__Helicobacteraceae;Other	0.00	0.00	0.00	0.00	0.359	0.508
f__Helicobacteraceae;g__	0.13	0.07	2.72	1.19	0.001	0.006
Flexispira	5.72	5.02	0.10	0.05	0.038	0.109
Helicobacter	7.75	6.81	4.75	3.91	0.500	0.642
f__Enterobacteriaceae;g__	0.01	0.01	0.02	0.01	0.225	0.367
f__F16;g__	0.05	0.05	0.16	0.10	0.024	0.076
Anaeroplasma	0.18	0.22	1.32	0.64	0.001	0.009
Mycoplasma	0.01	0.01	0.40	0.51	0.015	0.063
o__RF39;f__;g__	0.18	0.15	0.41	0.17	0.005	0.028
Akkermansia	0.03	0.02	2.56	4.63	0.083	0.201

Supplementary Table S2. Differences in bacterial taxa in 24 weeks-old wt mice treated or not treated with SLAB51

	not treated		treated SLAB51			
**Taxa**	**mean**	**SD**	**mean**	**SD**	**p-value**	**q-value**
**Phylum**						
Actinobacteria	0.06	18.28	0.34	0.33	0.058	0.146
Bacteroidetes	51.20	18.95	53.13	10.18	0.401	0.573
Cyanobacteria	0.05	12.46	0.02	0.02	0.018	0.059
Deferribacteres	0.00	13.12	0.06	0.13	0.002	0.012
Firmicutes	35.58	13.84	36.17	4.81	0.834	0.926
Proteobacteria	8.07	4.10	9.32	8.21	1.000	1.000
TM7	0.16	2.79	0.15	0.14	0.636	0.795
Tenericutes	2.13	3.20	0.59	0.55	0.002	0.012
Verrucomicrobia	2.56	4.63	0.05	0.05	0.399	0.573
**Class**						
Actinobacteria	0.00	0.00	0.25	0.29	0.040	0.094
Coriobacteriia	0.06	0.01	0.09	0.06	0.342	0.508
Bacteroidia	51.19	7.27	53.12	10.18	0.401	0.508
Sphingobacteriia	0.02	0.02	0.01	0.02	0.913	0.913
4C0d-2	0.04	0.03	0.01	0.01	0.017	0.054
Chloroplast	0.01	0.00	0.01	0.01	0.785	0.829
Deferribacteres	0.00	0.00	0.06	0.13	0.002	0.011
Bacilli	2.67	1.23	4.35	3.05	0.172	0.364
Clostridia	32.82	5.78	30.89	3.69	0.401	0.508
Erysipelotrichi	0.10	0.04	0.93	0.98	0.036	0.094
Alphaproteobacteria	0.21	0.17	0.18	0.14	0.674	0.754
Betaproteobacteria	0.07	0.04	0.68	0.61	0.005	0.017
Deltaproteobacteria	0.19	0.16	1.70	1.33	0.001	0.011
Epsilonproteobacteria	7.57	3.53	6.64	7.61	0.248	0.471
Gammaproteobacteria	0.03	0.01	0.12	0.06	0.002	0.011
TM7-3	0.16	0.10	0.15	0.14	0.636	0.754
Mollicutes	2.13	0.60	0.59	0.55	0.002	0.011
Verrucomicrobiae	2.56	4.63	0.05	0.05	0.399	0.508
**Order**						
Bifidobacteriales	0.00	0.00	0.25	0.29	0.018	0.052
Coriobacteriales	0.06	0.01	0.09	0.06	0.342	0.548
Bacteroidales	51.19	7.27	53.12	10.18	0.401	0.548
Sphingobacteriales	0.02	0.02	0.01	0.02	0.913	0.916
YS2	0.04	0.03	0.01	0.01	0.017	0.052
Streptophyta	0.00	0.01	0.01	0.01	0.585	0.737
Deferribacterales	0.00	0.00	0.06	0.13	0.002	0.020
Lactobacillales	2.64	1.23	2.82	1.78	0.916	0.916
Turicibacterales	0.02	0.01	1.52	1.97	0.023	0.060
Clostridiales	32.82	5.78	30.89	3.69	0.401	0.548
Erysipelotrichales	0.10	0.04	0.93	0.98	0.036	0.084
RF32	0.14	0.14	0.04	0.03	0.058	0.126
Rhizobiales	0.03	0.05	0.08	0.12	0.597	0.737
Rickettsiales	0.02	0.03	0.04	0.04	0.290	0.504
Sphingomonadales	0.01	0.01	0.01	0.01	0.652	0.737
Burkholderiales	0.07	0.04	0.68	0.61	0.005	0.023
Desulfovibrionales	0.19	0.16	1.70	1.33	0.001	0.020
Campylobacterales	7.57	3.53	6.64	7.61	0.248	0.496

Enterobacteriales	0.02	0.01	0.10	0.05	0.002	0.020
Pseudomonadales	0.00	0.00	0.01	0.01	0.011	0.049
CW040	0.16	0.10	0.15	0.14	0.636	0.737
Anaeroplasmatales	1.32	0.64	0.24	0.46	0.003	0.021
Mycoplasmatales	0.40	0.51	0.13	0.07	0.916	0.916
RF39	0.41	0.17	0.22	0.20	0.014	0.050
Verrucomicrobiales	2.56	4.63	0.05	0.05	0.399	0.548
**Family**						
Bifidobacteriaceae	0.00	0.00	0.25	0.29	0.018	0.059
Coriobacteriaceae	0.06	0.01	0.09	0.06	0.342	0.466
o__Bacteroidales;f__	0.01	0.01	0.73	0.48	0.001	0.012
Bacteroidaceae	4.04	1.69	4.28	1.21	0.834	0.852
Porphyromonadaceae	0.00	0.00	0.00	0.00	0.511	0.618
Prevotellaceae	0.07	0.10	0.00	0.00	0.128	0.246
Rikenellaceae	4.33	1.91	2.16	0.79	0.021	0.064
S24-7	37.13	6.31	39.93	8.46	0.462	0.591
[Odoribacteraceae]	5.59	2.07	6.00	2.01	0.713	0.763
[Paraprevotellaceae]	0.01	0.01	0.01	0.01	0.318	0.458
o__YS2;f__	0.04	0.03	0.01	0.01	0.017	0.059
o__Streptophyta;f__	0.00	0.01	0.01	0.01	0.585	0.690
Deferribacteraceae	0.00	0.00	0.06	0.13	0.002	0.018
Lactobacillaceae	2.64	1.23	2.72	1.72	0.674	0.739
Streptococcaceae	0.01	0.00	0.10	0.08	0.001	0.012
Turicibacteraceae	0.02	0.01	1.52	1.97	0.023	0.067
o__Clostridiales;Other	0.04	0.02	0.02	0.01	0.008	0.039
o__Clostridiales;f__	19.72	3.24	16.45	2.88	0.074	0.155
Christensenellaceae	0.00	0.00	0.01	0.01	0.034	0.086
Clostridiaceae	0.13	0.08	0.36	0.14	0.016	0.059
Dehalobacteriaceae	0.23	0.07	0.31	0.12	0.141	0.250
Lachnospiraceae	7.64	4.16	7.42	1.85	0.345	0.466
Peptococcaceae	0.02	0.01	0.04	0.02	0.066	0.143
Ruminococcaceae	5.02	1.12	6.26	2.21	0.142	0.250
[Mogibacteriaceae]	0.01	0.01	0.03	0.01	0.008	0.039
Erysipelotrichaceae	0.10	0.04	0.93	0.98	0.036	0.086
RF32;f__	0.14	0.14	0.04	0.03	0.058	0.134
Methylobacteriaceae	0.00	0.00	0.00	0.00	0.107	0.213
Phyllobacteriaceae	0.03	0.05	0.08	0.12	0.285	0.432
Rhizobiaceae	0.00	0.01	0.01	0.01	0.499	0.618
mitochondria	0.02	0.03	0.04	0.04	0.290	0.432
Sphingomonadaceae	0.01	0.01	0.01	0.01	0.652	0.732
Alcaligenaceae	0.05	0.03	0.66	0.61	0.008	0.039
Comamonadaceae	0.00	0.00	0.01	0.01	0.002	0.018
Oxalobacteraceae	0.01	0.01	0.01	0.01	0.748	0.782
Desulfovibrionaceae	0.19	0.16	1.70	1.33	0.001	0.012
Helicobacteraceae	7.57	3.53	6.64	7.61	0.248	0.407
Enterobacteriaceae	0.02	0.01	0.10	0.05	0.002	0.018
Moraxellaceae	0.00	0.00	0.01	0.01	0.031	0.083
Pseudomonadaceae	0.00	0.00	0.01	0.01	0.244	0.407
F16	0.16	0.10	0.15	0.14	0.636	0.732
Anaeroplasmataceae	1.32	0.64	0.24	0.46	0.003	0.022
Mycoplasmataceae	0.40	0.51	0.13	0.07	0.916	0.916
o__RF39;f__	0.41	0.17	0.22	0.20	0.014	0.056
Verrucomicrobiaceae	2.56	4.63	0.05	0.05	0.399	0.525

**Genus**						
Bifidobacterium	0.00	0.00	0.25	0.29	0.018	0.062
Coriobacteriaceae;g__	0.00	0.01	0.01	0.01	0.513	0.643
Adlercreutzia	0.05	0.01	0.08	0.06	0.342	0.505
o__Bacteroidales;Other;Other	0.01	0.01	0.73	0.48	0.001	0.014
Bacteroides	4.04	1.69	4.28	1.21	0.834	0.858
Parabacteroides	0.00	0.00	0.00	0.00	0.511	0.643
Prevotella	0.07	0.10	0.00	0.00	0.128	0.239
f__Rikenellaceae;g__	4.25	1.91	1.78	0.63	0.005	0.024
AF12	0.07	0.06	0.22	0.09	0.004	0.024
Rikenella	0.01	0.01	0.17	0.31	0.026	0.077
;f__S24-7;g__	37.13	6.31	39.93	8.46	0.462	0.613
Odoribacter	5.59	2.07	6.00	2.01	0.713	0.781
[Prevotella]	0.01	0.01	0.01	0.01	0.318	0.488
Pedobacter	0.01	0.02	0.01	0.01	0.822	0.858
o__YS2;f__;g__	0.04	0.03	0.01	0.01	0.017	0.062
o__Streptophyta;f__;g__	0.00	0.01	0.01	0.01	0.585	0.696
Mucispirillum	0.00	0.00	0.06	0.13	0.002	0.023
Lactobacillaceae;g__	0.00	0.00	0.00	0.00	0.027	0.077
Lactobacillus	2.64	1.23	2.72	1.72	0.674	0.751
Streptococcus	0.01	0.00	0.10	0.08	0.001	0.014
Turicibacter	0.02	0.01	1.52	1.97	0.023	0.073
o__Clostridiales;Other;Other	0.04	0.02	0.02	0.01	0.008	0.036
o__Clostridiales;f__;g__	19.72	3.24	16.45	2.88	0.074	0.165
f__Christensenellaceae;g__	0.00	0.00	0.01	0.01	0.011	0.043
f__Clostridiaceae;g__	0.04	0.02	0.08	0.07	0.317	0.488
Candidatus Arthromitus	0.01	0.01	0.05	0.05	0.051	0.130
Clostridium	0.08	0.07	0.23	0.18	0.172	0.304
Dehalobacterium	0.23	0.07	0.31	0.12	0.141	0.256
f__Lachnospiraceae;Other	0.13	0.05	0.10	0.05	0.344	0.505
f__Lachnospiraceae;g__	6.30	4.08	5.35	1.87	0.916	0.916
Anaerostipes	0.16	0.09	0.07	0.09	0.059	0.140
Coprococcus	0.53	0.21	1.02	0.29	0.002	0.023
Dorea	0.02	0.01	0.11	0.08	0.005	0.024
Roseburia	0.10	0.09	0.11	0.20	0.090	0.194
[Ruminococcus]	0.39	0.10	0.66	0.34	0.021	0.068
f__Peptococcaceae;g__	0.02	0.01	0.04	0.02	0.066	0.151
f__Ruminococcaceae;Other	0.01	0.01	0.01	0.00	0.746	0.799
f__Ruminococcaceae;g__	1.42	0.21	2.25	2.15	0.529	0.651
Oscillospira	2.36	0.46	2.82	1.10	0.401	0.553
Ruminococcus	1.23	0.95	1.18	0.52	0.600	0.701
f__[Mogibacteriaceae];g__	0.01	0.01	0.03	0.01	0.008	0.036
f__Erysipelotrichaceae;g__	0.08	0.04	0.19	0.12	0.103	0.209
Allobaculum	0.01	0.01	0.72	0.84	0.045	0.119
Coprobacillus	0.01	0.00	0.02	0.03	0.554	0.671
_RF32;f__;g__	0.14	0.14	0.04	0.03	0.058	0.140
Methylobacterium	0.00	0.00	0.00	0.00	0.117	0.225
Phyllobacterium	0.03	0.05	0.08	0.12	0.285	0.467
f__mitochondria;Other	0.02	0.03	0.04	0.04	0.290	0.467
Sutterella	0.05	0.03	0.66	0.61	0.008	0.036
Comamonadaceae;g__	0.00	0.00	0.01	0.01	0.003	0.024
f__Desulfovibrionaceae;g__	0.00	0.00	0.00	0.00	0.117	0.225
Bilophila	0.01	0.01	0.00	0.01	0.397	0.553

Oxalobacteraceae;g__	0.01	0.01	0.01	0.01	0.612	0.704
Desulfovibrionaceae;Other	0.02	0.01	0.01	0.01	0.424	0.574
Desulfovibrionaceae;g__	0.03	0.03	1.59	1.35	0.001	0.014
Desulfovibrio	0.14	0.15	0.10	0.07	0.493	0.642
f__Helicobacteraceae;Other	0.00	0.00	0.00	0.00	0.095	0.199
f__Helicobacteraceae;g__	2.72	1.19	3.75	3.95	0.753	0.799
Flexispira	0.10	0.05	2.76	3.74	0.001	0.014
Helicobacter	4.75	3.91	0.14	0.13	0.005	0.024
f__Enterobacteriaceae;g__	0.02	0.01	0.10	0.05	0.002	0.023
Acinetobacter	0.00	0.00	0.01	0.01	0.031	0.084
Pseudomonas	0.00	0.00	0.01	0.01	0.244	0.421
f__F16;g__	0.16	0.10	0.15	0.14	0.636	0.720
Anaeroplasma	1.32	0.64	0.24	0.46	0.003	0.024
Mycoplasma	0.40	0.51	0.13	0.07	0.916	0.916
o__RF39;f__;g__	0.41	0.17	0.22	0.20	0.014	0.052
Akkermansia	2.56	4.63	0.05	0.05	0.399	0.553

Supplementary Table S3. Differences in bacterial taxa in 24 weeks-old 3xTg-AD mice treated or not treated with SLAB51

	not treated		treated SLAB51			
**Taxa**	**mean**	**SD**	**mean**	**SD**	**p-value**	**q-value**
**Phylum**						
Actinobacteria	0.10	0.07	0.08	0.05	0.624	0.780
Bacteroidetes	41.78	16.82	60.46	13.92	0.028	0.138
Cyanobacteria	0.01	0.00	0.01	0.02	0.134	0.446
Deferribacteres	0.00	0.00	0.02	0.06	0.563	0.780
Firmicutes	42.99	15.25	36.93	13.55	0.568	0.780
Proteobacteria	14.52	11.52	1.86	0.94	0.028	0.138
TM7	0.05	0.05	0.04	0.04	0.219	0.548
Tenericutes	0.37	0.25	0.42	0.18	0.462	0.780
Verrucomicrobia	0.03	0.02	0.03	0.02	0.934	0.934
**Class**						
Actinobacteria	0.00	0.00	0.03	0.04	0.030	0.074
Coriobacteriia	0.10	0.07	0.05	0.03	0.191	0.375
Bacteroidia	41.77	16.82	60.45	13.92	0.028	0.074
4C0d-2	0.00	0.00	0.01	0.02	0.897	0.934
Chloroplast	0.01	0.00	0.00	0.00	0.007	0.042
Bacilli	3.63	5.57	5.49	7.64	0.744	0.903
Clostridia	39.16	14.36	31.12	13.34	0.221	0.375
Erysipelotrichi	0.20	0.14	0.31	0.23	0.438	0.655
Alphaproteobacteria	0.27	0.67	0.02	0.02	0.017	0.066
Betaproteobacteria	0.14	0.24	1.20	0.97	0.003	0.021
Deltaproteobacteria	0.48	0.39	0.39	0.35	0.653	0.854
Epsilonproteobacteria	13.60	11.89	0.13	0.11	0.003	0.021
Gammaproteobacteria	0.02	0.02	0.12	0.12	0.019	0.066
TM7-3	0.05	0.05	0.04	0.04	0.219	0.375
Mollicutes	0.37	0.25	0.42	0.18	0.462	0.655
Verrucomicrobiae	0.03	0.02	0.03	0.02	0.934	0.934

**Order**						
Bifidobacteriales	0.00	0.00	0.03	0.04	0.038	0.123
Coriobacteriales	0.10	0.07	0.05	0.03	0.191	0.383
Bacteroidales	41.77	16.82	60.45	13.92	0.028	0.102
YS2	0.00	0.00	0.01	0.02	0.758	0.934
Streptophyta	0.01	0.00	0.00	0.00	0.007	0.048
Bacillales	0.00	0.01	0.00	0.00	0.086	0.223
Lactobacillales	2.47	2.65	5.47	7.64	0.903	0.934
Turicibacterales	1.15	3.13	0.02	0.01	0.019	0.081
Clostridiales	39.16	14.36	31.12	13.34	0.221	0.383
Erysipelotrichales	0.20	0.14	0.31	0.23	0.438	0.711
Rhizobiales	0.23	0.63	0.01	0.01	0.113	0.266
Rickettsiales	0.03	0.04	0.01	0.01	0.047	0.137
Sphingomonadales	0.00	0.00	0.00	0.00	0.647	0.888
Burkholderiales	0.14	0.24	1.20	0.97	0.003	0.022
Desulfovibrionales	0.48	0.39	0.39	0.35	0.683	0.888
Campylobacterales	13.60	11.89	0.13	0.11	0.003	0.022
Aeromonadales	0.00	0.00	0.04	0.07	0.000	0.005
Enterobacteriales	0.01	0.01	0.07	0.07	0.010	0.051
Pseudomonadales	0.00	0.00	0.00	0.01	0.532	0.814
Xanthomonadales	0.00	0.01	0.00	0.01	0.855	0.934
CW040	0.05	0.05	0.04	0.04	0.219	0.383
Anaeroplasmatales	0.18	0.22	0.14	0.14	0.683	0.888
Mycoplasmatales	0.01	0.01	0.01	0.01	0.798	0.934
RF39	0.18	0.15	0.28	0.19	0.221	0.383
Verrucomicrobiales	0.03	0.02	0.03	0.02	0.934	0.934
**Family**						
Bifidobacteriaceae	0.00	0.00	0.03	0.04	0.038	0.227
Coriobacteriaceae	0.10	0.07	0.05	0.03	0.191	0.441
o__Bacteroidales;Other	0.00	0.00	0.00	0.00	0.179	0.441
;o__Bacteroidales;f__	0.09	0.09	0.45	0.70	0.540	0.756
Bacteroidaceae	2.41	3.09	3.70	2.16	0.191	0.441
Prevotellaceae	0.01	0.01	0.25	0.40	0.435	0.691
Rikenellaceae	3.88	2.51	5.75	4.94	0.514	0.744
S24-7	28.23	15.35	42.38	15.40	0.060	0.254
[Odoribacteraceae]	4.45	4.34	5.63	2.35	0.191	0.441
[Paraprevotellaceae]	2.69	3.03	1.82	2.82	0.086	0.301
o__YS2;f__	0.00	0.00	0.01	0.02	0.758	0.885
o__Streptophyta;f__	0.01	0.00	0.00	0.00	0.007	0.078
Enterococcaceae	0.04	0.10	0.00	0.00	0.051	0.245
Lactobacillaceae	2.40	2.66	5.45	7.64	1.000	1.000
Streptococcaceae	0.03	0.02	0.02	0.03	0.342	0.625
Turicibacteraceae	1.15	3.13	0.02	0.01	0.019	0.130
o__Clostridiales;Other	0.68	1.46	0.36	0.56	0.461	0.691
o__Clostridiales;f__	22.93	7.48	18.90	7.53	0.568	0.769
Christensenellaceae	0.01	0.01	0.01	0.01	0.376	0.658
Clostridiaceae	0.31	0.29	0.27	0.14	0.935	0.958
Dehalobacteriaceae	0.18	0.08	0.15	0.07	0.307	0.585
Lachnospiraceae	9.56	6.29	5.68	3.92	0.103	0.331
Peptococcaceae	0.04	0.05	0.04	0.06	0.682	0.819
Peptostreptococcaceae	0.03	0.06	0.00	0.00	0.068	0.260
Ruminococcaceae	5.37	1.65	5.68	2.67	0.870	0.937

[Mogibacteriaceae]	0.06	0.04	0.03	0.02	0.152	0.441
Erysipelotrichaceae	0.20	0.14	0.31	0.23	0.438	0.691
Phyllobacteriaceae	0.23	0.63	0.00	0.00	0.202	0.441
mitochondria	0.03	0.04	0.01	0.01	0.052	0.245
Sphingomonadaceae	0.00	0.00	0.00	0.00	0.647	0.819
Alcaligenaceae	0.13	0.24	1.19	0.97	0.002	0.035
Oxalobacteraceae	0.01	0.01	0.00	0.00	0.450	0.691
Desulfovibrionaceae	0.48	0.39	0.39	0.35	0.683	0.819
Helicobacteraceae	13.60	11.89	0.13	0.11	0.003	0.035
Aeromonadaceae	0.00	0.00	0.04	0.07	0.000	0.008
Enterobacteriaceae	0.01	0.01	0.07	0.07	0.010	0.082
F16	0.05	0.05	0.04	0.04	0.219	0.441
Anaeroplasmataceae	0.18	0.22	0.14	0.14	0.683	0.819
Mycoplasmataceae	0.01	0.01	0.01	0.01	0.798	0.906
o__RF39;f__	0.18	0.15	0.28	0.19	0.221	0.441
Verrucomicrobiaceae	0.03	0.02	0.03	0.02	0.934	0.958
**Genus**						
Bifidobacterium	0.00	0.00	0.03	0.04	0.038	0.151
Coriobacteriaceae;g__	0.00	0.01	0.01	0.02	0.233	0.415
Adlercreutzia	0.09	0.07	0.04	0.03	0.130	0.347
o__Bacteroidales;Other;Other	0.00	0.00	0.00	0.00	0.179	0.395
o__Bacteroidales;f__;g__	0.09	0.09	0.45	0.70	0.540	0.720
Bacteroides	2.41	3.09	3.70	2.16	0.191	0.395
f__Prevotellaceae;g__	0.01	0.01	0.01	0.02	0.523	0.713
Prevotella	0.00	0.00	0.24	0.38	0.178	0.395
f__Rikenellaceae;g__	3.67	2.38	4.47	3.18	0.744	0.898
AF12	0.09	0.21	0.47	0.81	0.105	0.292
Rikenella	0.12	0.17	0.81	1.62	0.740	0.898
;f__S24-7;g__	28.23	15.35	42.38	15.40	0.060	0.215
Odoribacter	4.45	4.34	5.63	2.35	0.191	0.395
[Prevotella]	2.69	3.03	1.82	2.82	0.086	0.262
o__YS2;f__;g__	0.00	0.00	0.01	0.02	0.758	0.899
o__Streptophyta;f__;g__	0.01	0.00	0.00	0.00	0.007	0.079
Enterococcus	0.04	0.10	0.00	0.00	0.027	0.146
Lactobacillus	2.40	2.66	5.45	7.64	1.000	1.000
Streptococcus	0.03	0.02	0.02	0.03	0.342	0.521
Turicibacter	1.15	3.13	0.02	0.01	0.019	0.129
o__Clostridiales;Other;Other	0.68	1.46	0.36	0.56	0.461	0.641
o__Clostridiales;f__;g__	22.93	7.48	18.90	7.53	0.568	0.741
f__Christensenellaceae;g__	0.01	0.01	0.01	0.01	0.376	0.560
f__Clostridiaceae;g__	0.09	0.08	0.16	0.10	0.086	0.262
Candidatus Arthromitus	0.10	0.13	0.04	0.05	0.190	0.395
Clostridium	0.11	0.16	0.07	0.09	0.837	0.940
SMB53	0.00	0.01	0.00	0.00	0.022	0.129
Dehalobacterium	0.18	0.08	0.15	0.07	0.307	0.491
f__Lachnospiraceae;Other	0.23	0.18	0.15	0.12	0.327	0.511
f__Lachnospiraceae;g__	7.56	5.75	4.52	3.78	0.142	0.362
Anaerostipes	0.01	0.01	0.02	0.05	0.286	0.473
Coprococcus	0.52	0.29	0.31	0.11	0.094	0.273
Dorea	0.09	0.04	0.07	0.03	0.413	0.600
Roseburia	0.00	0.00	0.01	0.02	0.230	0.415
[Ruminococcus]	1.15	0.56	0.60	0.33	0.022	0.129
f__Peptococcaceae;g__	0.04	0.05	0.04	0.06	0.682	0.857

f__Peptostreptococcaceae;g__	0.03	0.06	0.00	0.00	0.068	0.230
f__Ruminococcaceae;Other	0.01	0.01	0.02	0.03	0.901	0.961
f__Ruminococcaceae;g__	1.76	0.49	2.13	1.16	0.289	0.473
;g__Ruminococcus	1.46	0.79	0.82	0.43	0.037	0.151
f__[Mogibacteriaceae];g__	0.06	0.04	0.03	0.02	0.152	0.374
f__Erysipelotrichaceae;g__	0.11	0.06	0.13	0.11	1.000	1.000
Allobaculum	0.06	0.07	0.15	0.23	1.000	1.000
Coprobacillus	0.04	0.05	0.03	0.04	0.774	0.901
Phyllobacterium	0.23	0.63	0.00	0.00	0.202	0.404
f__mitochondria;Other	0.03	0.04	0.01	0.01	0.052	0.197
Sphingomonas	0.00	0.00	0.00	0.00	0.426	0.606
Sutterella	0.13	0.24	1.19	0.97	0.002	0.030
Desulfovibrionaceae;Other	0.34	0.31	0.11	0.17	0.034	0.151
f__Desulfovibrionaceae;g__	0.00	0.00	0.00	0.00	0.862	0.944
Bilophila	0.01	0.01	0.00	0.01	0.255	0.440
Desulfovibrio	0.13	0.25	0.27	0.28	0.037	0.151
f__Helicobacteraceae;Other	0.00	0.00	0.00	0.00	0.003	0.041
f__Helicobacteraceae;g__	0.13	0.07	0.05	0.01	0.010	0.091
Flexispira	5.72	5.02	0.01	0.01	0.002	0.030
Helicobacter	7.75	6.81	0.07	0.11	0.002	0.030
Aeromonadaceae;g__	0.00	0.00	0.04	0.06	0.000	0.013
f__Enterobacteriaceae;g__	0.01	0.01	0.06	0.06	0.017	0.129
f__F16;g__	0.05	0.05	0.04	0.04	0.219	0.415
Anaeroplasma	0.18	0.22	0.14	0.14	0.683	0.857
Mycoplasma	0.01	0.01	0.01	0.01	0.798	0.912
o__RF39;f__;g__	0.18	0.15	0.28	0.19	0.221	0.415
Akkermansia	0.03	0.02	0.03	0.02	0.934	0.980

Supplemental figure 1. Uncropped gels of blots presented in figures 5, 7 and 9. Cropped gels blots are reported when the PVDF membranes for Western Blot were cut into strips to minimize the amount of antibodies that are necessary for analysis.

Supplemental Fig 1 - Uncropped gels

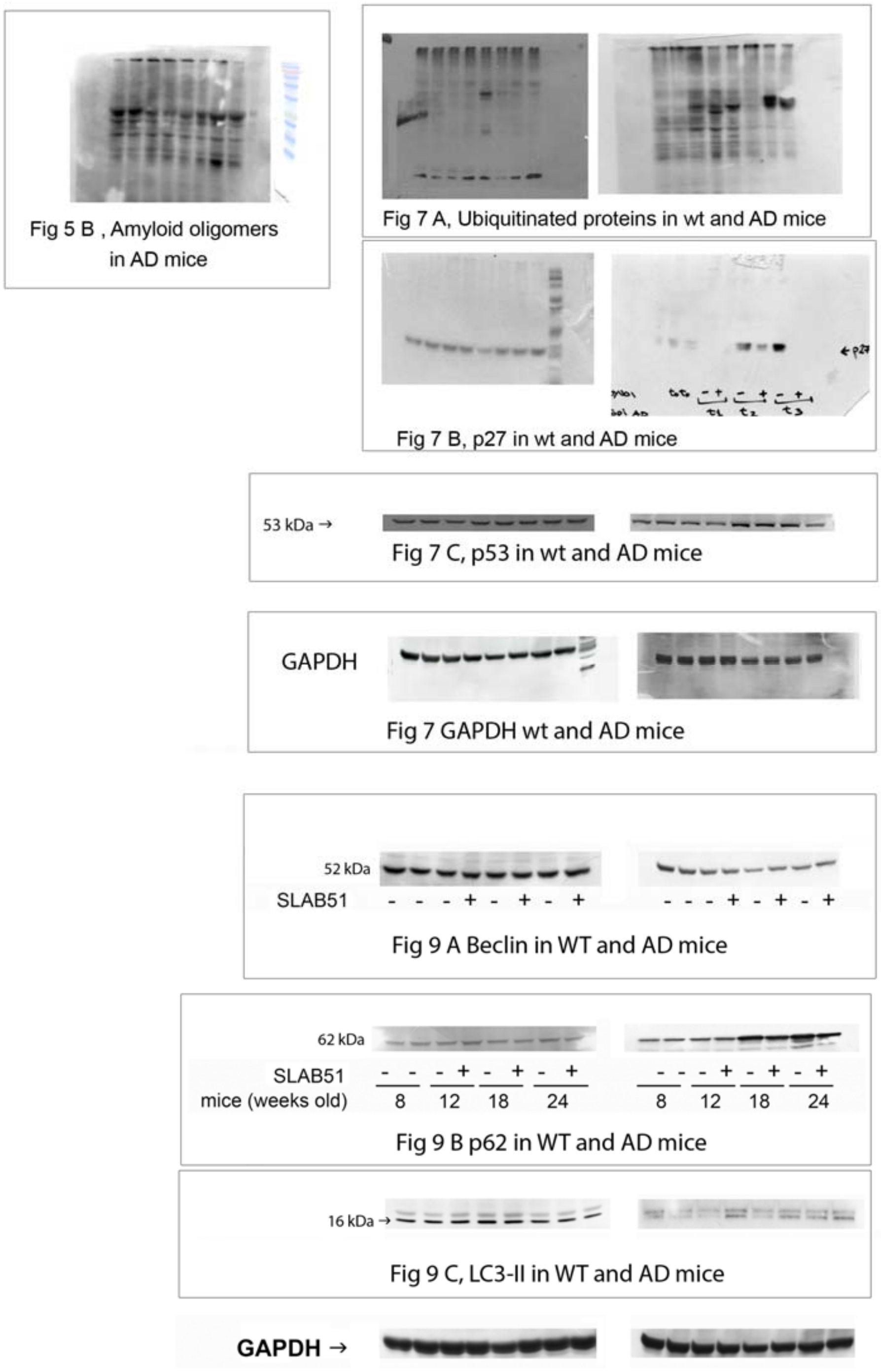

Supplement: Supplementary file 1 — Supplemental information [file 41598_2017_2587_MOESM1_ESM.pdf]
